# Mid-infrared photodetection with 2D metal halide perovskites at ambient temperature

**DOI:** 10.1126/sciadv.adk2778

**Published:** 2024-12-13

**Authors:** Yanyan Li, Shunran Li, Du Chen, Conrad A. Kocoj, Ankun Yang, Benjamin T. Diroll, Peijun Guo

**Affiliations:** ^1^ Department of Chemical and Environmental Engineering, Yale University, 9 Hillhouse Avenue, New Haven, CT 06520, USA.; ^2^ Energy Sciences Institute, Yale University, 810 West Campus Drive, West Haven, CT 06516, USA.; ^3^ Department of Mechanical Engineering, Oakland University, Rochester, MI 48309, USA.; ^4^ Center for Nanoscale Materials, Argonne National Laboratory, 9700 South Cass Avenue, Lemont, IL 60439, USA.

## Abstract

The detection of mid-infrared (MIR) light is technologically important for applications such as night vision, imaging, sensing, and thermal metrology. Traditional MIR photodetectors either require cryogenic cooling or have sophisticated device structures involving complex nanofabrication. Here, we conceive spectrally tunable MIR detection by using two-dimensional metal halide perovskites (2D-MHPs) as the critical building block. Leveraging the ultralow cross-plane thermal conductivity and strong temperature-dependent excitonic resonances of 2D-MHPs, we demonstrate ambient-temperature, all-optical detection of MIR light with sensitivity down to 1 nanowatt per square micrometer, using plastic substrates. Through the adoption of membrane-based structures and a photonic enhancement strategy unique to our all-optical detection modality, we further improved the sensitivity to sub–10 picowatt-per-square-micrometer levels. The detection covers the mid-wave infrared regime from 2 to 4.5 micrometers and extends to the long-wave infrared wavelength at 10.6 micrometers, with wavelength-independent sensitivity response. Our work opens a pathway to alternative types of solution-processable, long-wavelength thermal detectors for molecular sensing, environmental monitoring, and thermal imaging.

## Teaser

Two-dimensional metal halide perovskites are used for mid-infrared photodetection at ambient temperature.

## INTRODUCTION

The mid-infrared (MIR) spectral range, encompassing both mid-wave (MWIR, 3 to 5 μm) and long-wave (LWIR, 8 to 14 μm) infrared regions, is pivotal in numerous technological fields including gas and molecular sensing (*1*, *2*), bioimaging (*3*), environmental monitoring (*4*), free-space communication (*5*), and thermal energy harvesting (*6*). However, in stark contrast to the detection of visible and near-infrared light, MIR light detection presents unique challenges due to the inherently low photon energies involved. Traditional MIR photon detectors constructed from narrow-bandgap semiconductors, such as PbS, InSb, and HgCdTe, require cryogenic cooling to function effectively, which leads to increased size, weight, cost, and likelihood of device failure (*7*, *8*). The spectral responsivity of these detectors is constrained by the electronic bandgap of light-absorbing semiconductors. On the other hand, MIR thermal detectors, also known as bolometers, can operate at ambient temperature over a broader spectral range, at the expense of reduced detection bandwidth (*9*). Bolometers typically consist of a MIR light–absorbing element in close proximity to a temperature-sensitive resistive material, such as vanadium oxide (VO*_x_* with x ranging from 1.8 to 2) and amorphous silicon. To minimize thermal conductance between the heat-sensitive element and the support substrate, these detectors often incorporate complex device architectures that require multiple lithographic steps (*10*).

In recent years, several innovative approaches for MIR detection have emerged. For instance, a nanoparticle-on-resonator–based scheme was reported to convert 10-μm-wavelength light into visible light by leveraging strong interactions between molecular vibrational modes and plasmonic near-fields, achieving a sensitivity of 1 μW μm^−2^ (*11*). Another approach, which used lanthanide-based NaYF_4_:Nd@NaYF_4_ core-shell nanotransducers, exploits ratiometric luminescent properties to detect infrared radiation in the 4.5- to 10.8-μm range, with a detection limit of ~0.3 nW μm^−2^ (*12*). In addition, 1T-TaS_2_, a charge-density-wave material, has been shown for light detection from visible to terahertz wavelengths, with responsivities up to 300 pW Hz^−1/2^ (*13*). A further approach used plasmonic antennas coupled with hyperbolic phonon polaritons in hexagonal boron nitride (*h*-BN) to funnel MIR light onto a graphene p-n junction, enabling resonant detection in the 6.2- to 7.3-μm range with a sensitivity of 82 pW Hz^−1/2^ (*14*). In another report, a PtTe_2_/Si Schottky junction–based photodetector, built on a mosaic-like two-dimensional (2D) PtTe_2_ layer with ultrawide light absorption, has demonstrated light detection across a broad range from 0.2 to 10.6 μm (*15*). Despite these advancements, the quest for new materials and detection concepts that can improve speed and device scalability, while reducing fabrication costs, remains a high priority for MIR detection.

## RESULTS

### 2D perovskites for MIR detection

Metal halide perovskites (MHPs) have emerged as solution-processable semiconductors with promising applications in photovoltaics and optoelectronics (*16*–*22*). The strong electron-phonon interactions in MHPs lead to temperature-dependent variations in photoluminescence linewidths (*23*–*25*) and facilitate the formation of polarons, which dominate trapping, transport, and recombination of charge carriers in these materials (*26*, *27*). 2D MHPs represent a structurally and chemically diverse subclass of the MHP family with favorable optoelectronic properties (*28*–*30*). The charge-insulating organic-spacer cations in 2D-MHPs not only impose dielectric and quantum confinements on charge carriers (*31*–*33*), leading to the formation of excitons with spectrally narrow and strong optical absorption at room temperature, but also strongly impede heat flow along the cross-plane direction (*34*–*36*).

**Fig. 1.**
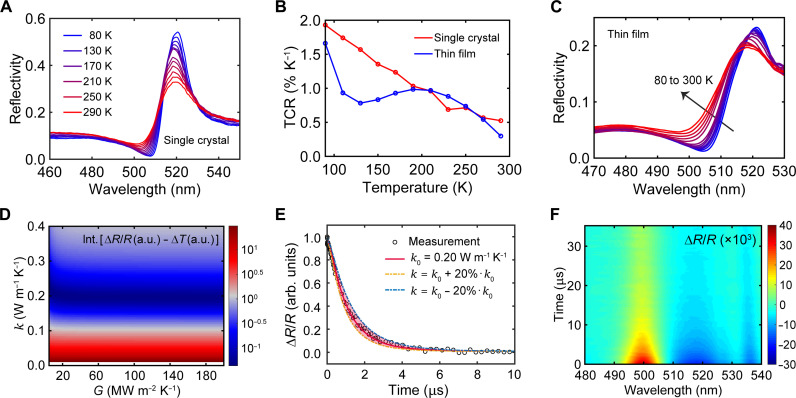
TCR and thermal transport properties of PEA. (**A**) Temperature-dependent reflectance spectra of PEA single crystal. (**B**) Temperature-dependent TCR at the reflectance-dip wavelength for PEA single crystal and spin-coated film. (**C**) Temperature-dependent reflectance spectra of a spin-coated PEA film with ~1-μm thickness. (**D**) Time-integrated difference between normalized ∆R/R at the reflectance-dip wavelength from IPVP experiments and normalized ∆T from finite-element simulations, shown as a function of κ and G. Note that the color bar is shown in log_10_ scale (i.e., bluer color indicates smaller fitting error). (**E**) Decay kinetics of ∆R/R at the reflectance-dip wavelength and the fitted decay of ∆T with finite-element simulations. The shaded area represents an 80% confidence band. The experimental data are taken from fig. S4. (**F**) Transient spectral map of ∆R/R for a 940-nm-thick PEA film on PET substrate measured at room temperature (pump wavelength, 3170 nm; fluence, 3.6 mJ cm^−2^).

While their ultralow thermal conductivity (denoted as κ) is unfavorable for 2D-MHPs in optoelectronic applications, here, we take advantage of this property as well as the strong temperature dependence of exciton-lattice interactions for a previously uncharted application of 2D-MHPs in thermal-type MIR photodetection. Our work demonstrates the viability of using 2D-MHPs as a previously unidentified and unique material platform for scalable, cost-effective MIR photodetection applications. Figure 1A shows the temperature-dependent reflectance spectra of (PEA)_2_PbI_4_ (abbreviated as PEA throughout this manuscript) single crystals, where PEA^+^ = C_6_H_5_C_2_H_4_NH_3_^+^, the phenylethylamine cation. PEA was chosen in this work because of its facile solution processability to yield high-crystallinity films and single-crystal membranes (*37*). The spectra were measured with unpolarized light at normal incidence on the crystalline *ab* plane or the (001) plane, which is parallel to the octahedral layers. The exciton resonance is manifested as a peak in reflectance near 520 nm and a dip on the shorter-wavelength side. Such a spectral feature in reflectance can be quantitatively captured by modeling the exciton resonance using a Lorentzian oscillator, with which the relative permittivity at angular frequency ω is written as $\varepsilon \left(\omega \right)=\varepsilon_{\infty }+\frac{{A_{L}}^{2}}{{\omega_{L}}^{2}-\omega^{2}-i\omega \gamma }$ (*38*–*40*). By fitting the reflectance spectrum at room temperature, we obtained the high-frequency permittivity $\varepsilon_{\infty }=5.07$, the oscillator strength $A_{L}=1.15\;\text{eV}$, the exciton energy $\omega_{L}=2.39\;\text{eV}$, and the damping factor γ=67 meV (fig. S1).

As shown in Fig. 1A, the resonant feature in reflectance strengthens with decreasing temperature, which results in a further increase (decrease) of reflectance at the reflectance peak (dip) (*41*, *42*). This trend arises from reduced electron-phonon interactions at lower temperatures, leading to homogeneous exciton linewidth narrowing. Notably, the low reflection value at the reflectance dip (500 to 510 nm) and its spectral shift with temperature yield a large temperature coefficient of reflectance (TCR), defined as the relative change in reflectance per degree change in temperature: $\text{TCR}\left(T\right)=\frac{R\left(T+\mathrm{dT}\right)-R\left(T\right)}{R\left(T\right)\cdot \mathrm{dT}}$, where T is the temperature and R is the temperature-dependent reflectance. As shown in Fig. 1B, at ambient temperature, the TCR is 0.52% K^−1^, which goes up to 2% K^−1^ at liquid-nitrogen temperature. The wavelength of maximal TCR slightly varies with temperature because of the spectral shift of the reflectance dip (fig. S2).

Similar to other *n* = 1 2D-MHPs (*30*), spin-coated PEA films exhibit a high degree of out-of-plane alignment (i.e., the *ab* crystalline planes are aligned parallel to the substrates), as verified with x-ray diffraction (XRD; fig. S3). Figure 1C shows the temperature-dependent reflectance spectra for a 1.0-μm-thick spin-coated PEA film. Because the thickness is larger than the optical penetration depth around the exciton resonance (fig. S1D), the film is considered infinitely thick, and the reflectance is substrate-blind. The film exhibits a TCR of ~0.3% K^−1^ at ambient temperature (Fig. 1B). The lower TCR of the film, as well as the slightly different temperature dependence of the exciton resonance, compared to its single-crystal analog likely arises from disordered in-plane crystallographic orientations (*43*), strain imposed by the substrate, and defects, which yield additional exciton damping pathways as evident from the broadened resonance in the reflectance spectra (Fig. 1C). The sizeable exciton-enabled TCR of PEA single crystals and films offers an opportunity for all-optical MIR photodetection, where the MIR light–induced temperature rise of PEA is detected by measuring the relative change in reflectance at the reflectance dip where the TCR is at its maximum.

Strong light-induced temperature effects are favored for the proposed MIR detection and are generally observed in materials with low κ. Because of the high density of organic-inorganic interfaces and the weak van der Waals forces connecting the organic cations between neighboring layers (*34*), 2D-MHPs have some of the lowest κ among extended solids, especially along the cross-plane direction. To determine the cross-plane κ of PEA at ambient temperature, we performed infrared-pump visible-probe (IPVP) transient reflection (TR) experiments for a 640-nm-thick PEA film deposited on single-crystalline Si wafer. The PEA was vibrationally (i.e., thermally) excited by MIR pulses centered at 3170 nm (resonant with the N─H and C─H stretching modes), and the pump-induced reflectance change was captured by a time-delayed, broadband visible probe (*44*). Because the pump photon energy is much lower than the electronic bandgap, the relevant process is a solely pump-induced, impulsive lattice heating, followed by its subsequent dissipation. The measured ΔR/R transient spectral map is shown in fig. S4, where ΔR/R=[R(t)−R(0)]/R(0) is the differential change in reflectance with R(0) being the reflectance before pump excitation and R(t) the reflectance at delay time t after pump excitation. The ΔR/R spectra exhibit a derivative-like line shape, transitioning from positive to negative at a zero-crossing wavelength of ~507 nm. The ΔR/R spectra, when compared with the steady-state data (Fig. 1, A and C), indicate impulsive pump-induced lattice heating followed by slow cooling in the microsecond timescale. Because lattice cooling typically takes nanoseconds (e.g., as measured in time-domain thermoreflectance) (*45*), the microsecond cooling time here reveals an ultralow cross-plane κ of PEA.

Using the finite-element method (see Materials and Methods), we simulated the heat-transfer process in PEA/Si with PEA subject to impulsive thermal excitation. The initial temperature profile in PEA was determined by its absorption coefficient at the MIR pump wavelength. A linear relationship was assumed between the measured ΔR/R and the simulated temperature rise (denoted as ΔT, averaged over the probe’s penetration depth). By sweeping the κ of PEA and the interfacial thermal conductance (G) between PEA and Si, we calculated the time-dependent ΔT and with it the time-integrated differences between the normalized decays of ΔR/R and ΔT. The obtained difference map (Fig. 1D) distills $\kappa =0.20\;W\;m^{-1}\;K^{-1}$ for PEA (assuming $G>20\;\text{MW}\;m^{-2}\;K^{-1}$). The sensitivity analysis in Fig. 1E indicates that the estimated κ of PEA should lie within ±20% of the true value.

Having determined the κ, we can then calculate the figure of merit, which we define as $\text{TCR}/\left(\kappa \cdot C_{p}\right)$, to quantify material performance for bolometer applications. The figure of merit is defined as $\text{TCR}/\left(\kappa \cdot C_{p}\right)$ because a higher TCR, a lower κ, and a lower $C_{p}$ all contribute to improved MIR light–induced material response. As presented in Table 1, PEA exhibits a figure of merit an order of magnitude higher than current state-of-the-art bolometer material VO_*x*_ (*46*, *47*). The sizeable TCR and exceptional heat retention of 2D-MHPs motivated us to repurpose these materials for MIR photodetection.

**Table 1.** A comparison between the state-of-the-art bolometer material VO_*x*_ and PEA. The figure of merit is defined as $\text{TCR}/\left(\kappa \cdot C_{p}\right)$ with a dimension of 10^−6^ kg·m·s·K^2^. *R* in TCR denotes resistivity for VO_*x*_ and reflectance for PEA. All properties are for room temperature.

Material	κ (W m^−1^ K^−1^)	TCR (% K^−1^)	*C*_*p*_ (J g^−1^ K^−1^)	Figure of merit
VO_*x*_	3.5~6	2	0.65	5–9
PEA	0.20	0.52	0.46	57

### Interfacing PEA with MIR-absorbing nanoantennas

To enable photodetection, we first used plastic polyethylene terephthalate (PET) as the underlying substrate to enhance heat retention within the PEA film, leveraging the low κ of PET ($<0.20\;W\;m^{-1}\;K^{-1}$). The transient ΔR/R spectral map from IPVP experiments for a 940-nm-thick PEA film spin-coated on a PET substrate is shown in Fig. 1F. The slow decay of ΔR/R takes tens of microseconds (i.e., an order of magnitude longer than PEA/Si in Fig. 1E), suggesting effective heat retention within the PEA film. However, similar to VO_*x*_, 2D-MHPs are transparent to MIR light except at discrete vibrational frequencies of the organic spacers. Strong MIR light absorbers need to be placed in proximity to 2D-MHPs, preferably away from the substrate, to enable the MIR detection functionality. To this end, localized surface plasmon resonances (LSPRs) provide a strong optical absorption cross section with subwavelength material volumes. We harvest LSPRs of indium-doped cadmium oxide (ICO), a type of transparent conducting oxide (TCO) with low optical loss and tunable free electron concentration (*48*–*50*). Besides providing MIR absorption, another important advantage of TCOs is visible transparency, making them compatible with the all-optical detection scheme here.

**Fig. 2.**
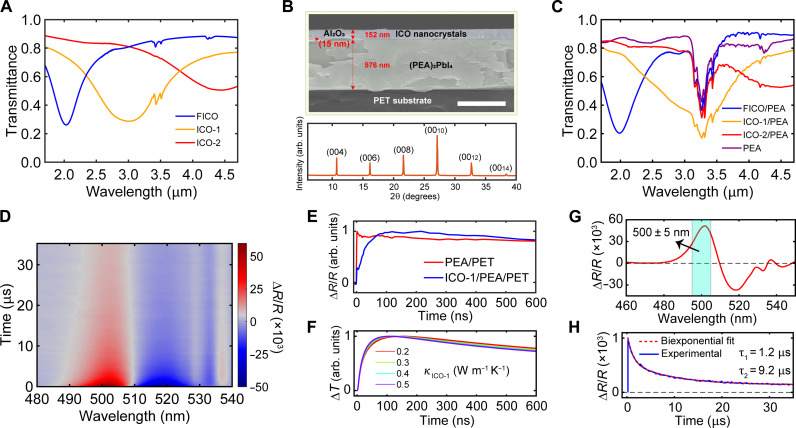
Optical and structural characterization of PEA films coated with ICO NCs. (**A**) MIR transmittance spectra of ICO NC films with different doping concentrations on sapphire substrates. (**B**) Cross-sectional SEM image of ICO-1/PEA/PET (scale bar, 1 μm) and its XRD pattern (the out-of-plane XRD peaks of PEA are indexed). (**C**) MIR transmittance spectra of CaF_2_ substrate–supported PEA films with and without ICO NC films on top. (**D**) Transient ∆R/R spectral map of ICO-1/PEA/PET at room temperature (pump wavelength, 3170 nm; fluence, 3.9 mJ·cm^−2^). (**E**) Comparison of ∆R/R kinetics at 500 nm between PEA/PET and ICO-1/PEA/PET. (**F**) Simulated decays of ∆T for PEA (averaged for the top 140 nm, which is the probe penetration depth) in ICO-1/PEA/PET, obtained with various values of κ for the ICO-1. (**G**) Transient ∆R/R spectrum at 100 ns and (**H**) ∆R/R kinetics at 500 nm and its biexponential fit; data are taken from (D).

We synthesized colloidal nanocrystals (NCs) of ICO with a high size uniformity by established ramped heating methods (*51*). By adjusting the dopant concentrations, three different NCs were prepared, and with a decreasing doping concentration, NCs dissolved in carbon tetrachloride exhibit LSPRs centered at 1.6, 2.4, and 3.4 μm (fig. S5). Because of increased interparticle interactions and higher dielectric constants of the surrounding medium, spin-casted NC films show red-shifted LSPRs centered at 2.0, 3.0, and 4.4 μm (Fig. 2A); we denote these NCs as FICO (fluorine- and indium-doped cadmium oxide), ICO-1 (indium-doped cadmium oxide-1), and ICO-2, respectively. We spin-coated NC films onto the PEA/PET stacks. A 15-nm, visibly transparent Al_2_O_3_ layer was pre-evaporated on the PEA/PET to prevent PEA from damage by toluene, the solvent of the ICO NCs. Figure 2B shows a cross-sectional scanning electron microscopy (SEM) image of the ICO-1/PEA/PET stack; the corresponding XRD pattern illustrates the structural integrity of the PEA film following the NC deposition. MIR transmittance spectra of the three types of ICO/PEA/PET samples are plotted in Fig. 2C. Notably, the absorption of MIR light by ICO NCs is largely preserved for the stacks, indicating the effectiveness of LSPR-enabled, spectrally tunable MIR absorption. Note that the LSPR absorption of ICO-1 overlaps with, but is much broader than, the N─H stretching absorption of PEA.

The transient ΔR/R spectral map (Fig. 2D) for the ICO-1/PEA/PET sample, pumped at 3170 nm, displays a similarly slow timescale as the PEA/PET (Fig. 1F). However, the ΔR/R of ICO-1/PEA/PET undergoes a slower rise (~100 ns) after time zero, whereas the ΔR/R of PEA/PET rises instantaneously following pump excitation (Fig. 2E and fig. S6). Such disparity is also observed for samples on Si substrates (fig. S7). Because of the visible transparency of ICO, the ΔR/R at 500 nm primarily arises from the temperature increase of PEA; hence, the 100-ns rise of ΔR/R can be ascribed to the NC-to-PEA heat transfer (fig. S8). Given a lack of knowledge on the κ for the NC film, we used several different κ for the NC film to simulate heat transfer from NC to PEA using the finite-element method and plotted the ΔT for PEA (averaged over the probe penetration depth). The range of κ (0.2 to 0.5 W m^−1^ K^−1^) in these simulations is informed by thermal-transport studies on semiconductor NC films, which found similar κ values for a wide range of NC solids (*52*). We find that the ΔT for PEA is largely independent of the κ of the NC film (Fig. 2F), and the simulated temporal profiles of temperature rise match well with the measured kinetics of ΔR/R. Inspection of the ΔR/R spectrum (Fig. 2G) shows that the maximal TCR at room temperature is centered at ~500 nm. A biexponential fit to the 500-nm ΔR/R kinetics distills exponential time constants of 1.2 and 9.2 μs in the 35-μs time window (Fig. 2H).

### Spectrally tunable MIR detection with PET-supported PEA

**Fig. 3.**
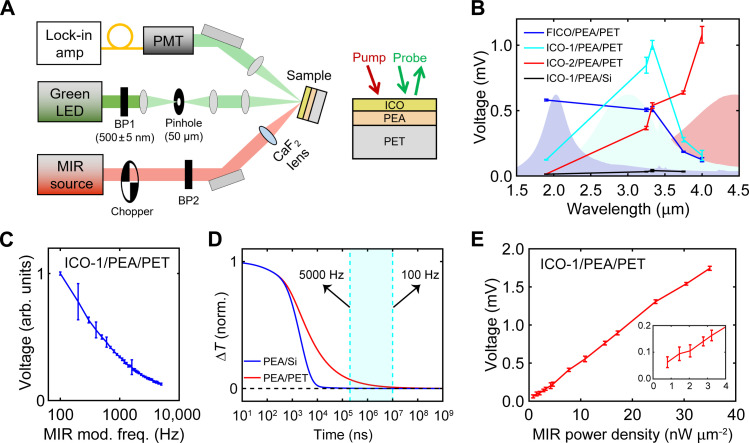
Spectrally tunable, all-optical MIR photodetection using ICO/PEA/PET stacks. (**A**) Schematic depiction of the all-optical MIR photodetection setup; BP1 (BP2) is the band-pass filter for the visible probe (the MIR pump). (**B**) Lock-in voltage for PEA/PET coated with the three different ICO-NC films; data for PEA/Si coated with ICO-1 measured at the same MIR power density are also shown for comparison. Shaded regions correspond to ICO NC absorbance in arbitrary units. (**C**) Lock-in voltage of ICO-1/PEA/PET as a function of the chopper frequency. (**D**) Finite-element simulated decays of the temperature averaged over the probe penetration depth (140 nm) of a 940-nm-thick PEA film deposited on Si and PET substrates. (**E**) Lock-in voltage of ICO-1/PEA/PET versus MIR (3.33 μm) power density (inset shows the range from 0 to 4 nW μm^−2^).

With an understanding of the optical and thermal-transport properties of the ICO/PEA/PET stack, we designed and executed all-optical MIR photodetection experiments, as depicted in Fig. 3A. A band-pass filter of 500 ± 5 nm defined a monochromatic probe at a wavelength where the sample exhibits the highest TCR. A broadband quasi–continuous-wave laser served as the MIR source, and five different MIR band-pass filters (fig. S9) were used to define the wavelengths of MIR light incident on the sample. The MIR light was modulated by a mechanical chopper to enable lock-in detection of the variation in the reflected probe intensity. Figure 3B summarizes the all-optical MIR detection results for PEA/PET coated with the three different ICO NCs measured at ambient temperature. Consistent with LSPR-enabled absorption (Fig. 2C and fig. S10), the sensitivities of the samples all peak at the LSPR maxima wavelengths of the corresponding NC films. On top of this, all the samples show photo-responses at 3 to 3.5 μm, which arise from N─H stretching absorption of PEA. As summarized in fig. S11, the measured lock-in voltage is linearly proportional to the incident MIR power, indicating a linear operational regime. Films on PET substrates show a sensitivity an order of magnitude higher than those on Si (Fig. 3B), which is in line with enhanced thermal retention with PET substrates (fig. S4 versus Fig. 2D). The millivolt-level lock-in voltage is a few thousandths of the photomultiplier tube (PMT) total output voltage (~0.5 V), from which we can estimate a relative change in reflectance to be less than 1% and hence a temperature modulation on the order of 1 K.

By varying the chopping frequency, we find the lock-in voltage increases with a decreasing chopping frequency over the explored range of 5000 to 100 Hz (Fig. 3C). Such a monotonic relationship implies a long thermal time constant of at least 10 ms for the ICO/PEA/PET stacks. Thermal-transport simulations with impulsive heating at time zero (Fig. 3D) reveal that although the ∆T of PEA undergoes substantial decay by 100 μs (i.e., in accordance with the IPVP results in Fig. 2H), the ∆T experiences a longer-lived tail. The thermal accumulation effect stemming from the long-lived component underpins the higher lock-in voltage achieved at lower chopping rates, and these results indicate that higher sensitivity can be achieved by further reducing the chopping rate at a cost of the detection bandwidth. To test the lowest MIR detection limit, we examined an ICO-1/PEA/PET sample under variable MIR powers at a wavelength of 3.33 μm with 100 Hz of MIR modulation frequency. As shown in Fig. 3E, the smallest detectable power density, which we define as when the signal is equivalent to the noise, reaches below 1 nW μm^−2^. The ratio of the smallest detectable lock-in voltage (0.07 mV) and the PMT voltage (0.5 V) corresponds to a ∆R/R of ~0.014%.

### Sensitivity enhancement by membrane-based structures

**Fig. 4.**
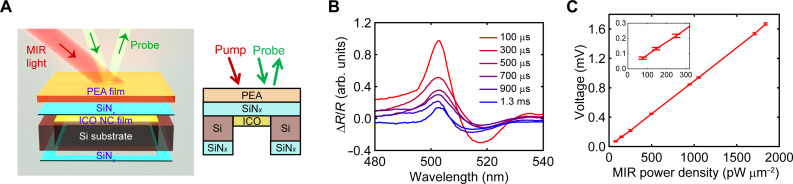
Thin membrane–based structure exhibits < 100 pW μm^−2^ sensitivity. (**A**) Schematic illustration of the PEA/SiN*_x_*/ICO-1 membrane structure supported by a Si substrate (left: 3D illustration; right: 2D view). (**B**) Transient ∆R/R spectra of a floating PEA membrane for varying delay times (100 μs to 1.3 ms). (**C**) Lock-in voltage of PEA/SiN*_x_*/ICO-1 on Si versus MIR (3.33 μm) power density (inset shows the range from 0 to 300 pW μm^−2^).

To further enhance the sensitivity to levels below the nanowatt-per-square-micrometer range, we then adopted a membrane-based structure leveraging the anisotropic, wet chemical etching properties of silicon. The rationale behind this design is that a membrane-based architecture can suppress vertical heat flow channels and hence slow down the overall heat dissipation rate. Figure 4A shows a schematic of the obtained PEA/SiN*_x_*/ICO structure. As illustrated in fig. S12, the process begins with a commercially obtained low-stress SiN*_x_*-coated Si wafer. For reasons described later, the thickness of SiN*_x_* was chosen to be 265 nm throughout the work. Using the well-established KOH wet-chemical etching method, we created several-hundred-micrometer-sized SiN*_x_* membrane windows on Si substrates. A film of PEA was then spin-coated onto the SiN*_x_* membrane, followed by the application of an ICO NC film, which was spin-coated onto the reverse side of the SiN*_x_* membrane. We then evaluated the heat dissipation rates of the membrane structure through TR experiments. As shown in Fig. 4B, the decay of ∆R/R occurs over a duration exceeding 1 ms. This level of heat retention, much more effective than that of PET substrate–supported stacks (Fig. 2, D and H), contributes to a notable improvement in MIR detectivity. As illustrated in Fig. 4C, we achieved an order-of-magnitude enhancement in detectivity, reaching the tens of picowatt-per-square-micrometer range, all while maintaining a fixed MIR modulation frequency of 80 Hz.

**Fig. 5.**
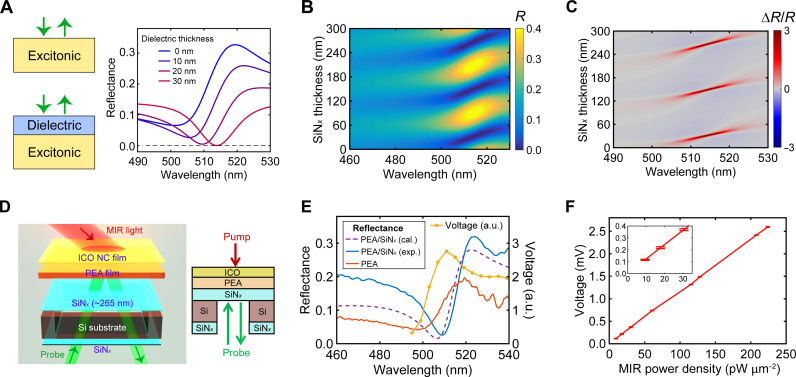
Thin membrane–based structure incorporating a dielectric coating enables <10 pW μm^−2^ sensitivity. (**A**) Demonstration of the dielectric coating–induced decrease in reflectance near the excitonic resonance of PEA. (**B**) Transfer matrix–calculated reflectance spectra of PEA film covered by SiN*_x_* (refractive index n=2.19) with SiN*_x_* thickness varied from 0 to 300 nm. (**C**) Color-coded map of $∆R/R=\left(R_{\gamma =77\;\text{meV}}-R_{\gamma =67\;\text{meV}}\right)/R_{\gamma =67\;\text{meV}}$ plotted as a function of wavelength and SiN*_x_* thickness. Here, γ=67 meV is the fitted Lorentzian damping factor for PEA at ambient temperature (fig. S1), and, without the loss of generality, the increase of γ from 67 to 77 meV is assumed to be induced by MIR light irradiation. (**D**) Schematic drawing of the inverted ICO/PEA/SiN*_x_* structure on Si substrate and the associated measurement scheme (left: 3D illustration; right: 2D view). (**E**) Reflectance spectra for PEA covered with a 265-nm-thick SiN*_x_* (calculated, purple-dashed; measured, blue) and for PEA without any SiN*_x_* coverage (red, taken from data in Fig. 1C). The measured lock-in voltage as a function of probe wavelength is shown in yellow for a PEA covered with a 265-nm-thick SiN*_x_*. (**F**) Lock-in voltage of ICO-1/PEA/SiN*_x_* versus MIR (3.33 μm in wavelength) power density (inset shows the range from 0 to 35 pW μm^−2^).

To further enhance the sensitivity, we focus on the advantage of our all-optical detection scheme here, which permits the design of photonic structures to potentially yield a larger magnitude of ∆R/R in response to a fixed, MIR light–induced temperature change in the PEA layer. Specifically, we exploited the nontrivial phase shift that occurs at the interface between the optically lossy PEA, especially near its exciton resonance, and a lossless dielectric material. As demonstrated in Fig. 5A, a thin dielectric coating onto the PEA enables enhanced absorption within PEA even with dielectric layers being considerably thinner than a quarter wavelength (λ/4) (*53*). For illustrating this concept, we used the transfer-matrix approach to calculate the reflectance spectra of PEA covered by a dielectric layer of varying thickness, using SiN*_x_* as a model dielectric material. The calculated reflectance map in Fig. 5B shows that the interference effects caused by the phase shift at the PEA/SiN*_x_* interface drastically alter the reflectance characteristics near the exciton resonance of PEA. The data further reveal that the changes in reflectance near the exciton resonance are periodic in the coating thickness and, crucially, for certain SiN*_x_* coating thicknesses, specifically ~30, 150, and 270 nm, the reflectance can approach nearly zero, corresponding to nearly complete optical absorption by the PEA.

A low steady-state reflectance (*R*) is advantageous for our MIR detection scheme. This is because, by comparing the ∆R/R map in Fig. 5C and the reflectance in Fig. 5B, we find that a lower reflectance *R* leads to an increased relative change in reflectance (∆R/R, where steady-state reflectance serves as the denominator). The ∆R/R is a critical factor in our signal readout for a fixed increase in exciton damping (∆γ) or, equivalently, a fixed temperature rise in the PEA layer. Theoretically, as the steady-state reflectance approaches zero, the value of ∆R/R tends to diverge. However, in practice, limitations to this divergence in enhancement exist, including those arising from the interfacial roughness between PEA and SiN*_x_*, nonuniformity of the PEA film, and the thickness variation of the SiN*_x_* layer, placing a ceiling on the maximum achievable MIR sensitivity. Despite these practical constraints, this dielectric-coating strategy still holds notable promise to further improve the sensitivity of our platform beyond the tens of picowatt-per-square-micrometer range.

While the dielectric layer depicted in Fig. 5A can be deposited onto PEA using straightforward physical vapor deposition techniques, we found an intriguing alternative by treating the PEA-supporting SiN*_x_* membrane itself as the dielectric layer. Notably, this approach is viable when the probe is incident on the SiN*_x_* side rather than directly on the PEA. To accommodate this, we slightly modified our device structure used in Fig. 4. The new structure, ICO/PEA/SiN*_x_*, and the associated configuration for MIR detection is depicted in Fig. 5D (see fig. S12 for sample fabrication details). We chose the SiN*_x_* thickness to be 265 nm because it is close to the condition where ∆R/R reaches a high value (Fig. 5C). The measurements on ICO/PEA/SiN*_x_* use a counter-propagating configuration, where the MIR light and visible probe light shine on the sample from opposing directions. Consistent with the transfer-matrix calculations, our measurements, as summarized in Fig. 5E, showed that when covered with the 265-nm-thick SiN*_x_*, the PEA displays a lower reflectance at its resonance dip and, importantly, a higher quality factor (i.e., sharper reflectance dip), when compared to an uncovered PEA film, all measured at room temperature. The MIR sensitivity, manifested as the measured lock-in voltage in Fig. 5E, reaches a maximum when using a probe wavelength matching the reflectance dip. We tested the MIR sensitivity of such dielectric layer–enhanced device by using ICO-1 as the MIR absorber. MIR power–dependent measurements (Fig. 5F), using a probe with 8-nm full width at half maximum in spectral bandwidth and a MIR pump centered at 3.33 μm, indicate that our structure achieves a remarkable sensitivity below 10 pW μm^−2^, nearly two orders of magnitude more sensitive than a previous report using ratiometric luminescence (*12*).

**Fig. 6.**
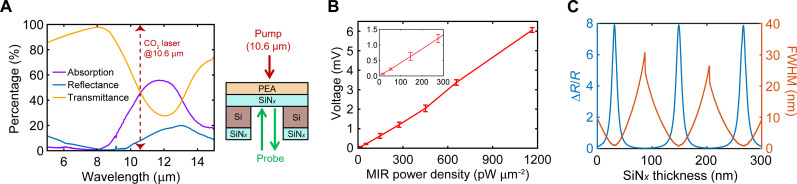
LWIR detection at 10.6-μm wavelength. (**A**) Left: Experimental transmittance, experimental reflectance, and calculated absorption for a 265-nm-thick SiN*_x_* membrane from 5 to 15 μm. Right: Schematic drawing of the sample and measurement configuration. (**B**) Lock-in voltage of PEA/SiN*_x_* versus MIR (10.6 μm in wavelength) power density (inset shows the range from 0 to 300 pW μm^−2^). (**C**) The maximum value of ∆R/R as a function of the thickness of the SiN*_x_* layer on top of PEA, and the associated full width at half maximum (FWHM) of the peak of ∆R/R, both calculated from the transfer-matrix calculated data in Fig. 5C.

To demonstrate the versatility of our approach, we expanded the detection from the MWIR range of 2 to 4.5 μm to the LWIR range at 10.6 μm, using a continuous-wave CO_2_ laser as the MIR excitation source. Here, we used a simpler SiN*_x_*/PEA bilayer structure, excluding the ICO NC layer. As shown in Fig. 6A, the infrared-active Si-N stretching mode of the 265-nm-thick SiN*_x_* film leads to a broad and efficient vibrational absorption centered between 10 and 13 μm (*54*), achieving ~50% absorption at 10.6 μm. A MIR sensitivity of 15.6 pW μm^−2^ at this wavelength was achieved (Fig. 6B), which is comparable to the performance observed in the MWIR regime (i.e., 2~5 μm). Looking forward, we anticipate that the sensitivity of our all-optical scheme could be further improved by optimizing the SiN*_x_* layer thickness (i.e., better matching the conditions that produce maximum enhancement of ∆R/R in Fig. 5C), increasing the interfacial contact between SiN*_x_* and PEA (potentially by adopting single-crystalline membranes), and simultaneously using a probe light of a narrower spectral bandwidth with full width at half maximum of 1 to 2 nm. The last point is further illustrated in Fig. 6C, where stronger ∆R/R occurs at discrete, precise thicknesses of SiN*_x_* when measured with a spectrally narrow probe. Cryogenic operation can also enable larger sensitivity due to a higher TCR of PEA at lower temperatures (Fig. 1B and figs. S13 and S14).

## DISCUSSION

In summary, leveraging the ultralow κ and the large exciton-induced TCR (two properties rarely available together) of 2D-MHPs, we demonstrated a previously uncharted application of these materials in MIR thermal detection using PEA as a prototypical 2D-MHP. A comparison table (table S1) shows that our proof-of-concept, 2D-MHP–based scheme compares favorably with other emerging methods for broadband MIR detection unburdened by materials’ bandgaps. Stability testing, as summarized in fig. S16, shows that if the PEA film’s back side is covered by a thin but compact dielectric layer, samples can be stable over several hours under a MIR power density two to three orders of magnitude larger than the detection limit. However, direct exposure of PEA’s back side to ambient air leads to sample degradation within a few tens of minutes under similar MIR power intensities. Looking beyond our current material of choice, other emerging hybrid layered materials might offer even lower thermal conductivity and higher TCR, leading to further advancements in MIR detectivity (*55*, *56*). The concept demonstrated here can be extended toward realizing MIR imagers with lithographically defined 2D arrays of SiN_x_ membrane windows. Furthermore, our approach can be adapted for multiplexed MIR detection, capturing both intensity and wavelength information, by integrating pixelated plasmonic nanoantennas or by using TCO NC pads with varying doping concentrations using ink-jet printing or other NC patterning techniques. The scope of light detection can be expanded into the far-infrared and terahertz range when 2D-MHPs are interfaced with optical resonators exhibiting strong plasmonic or phononic absorption at those spectral ranges (*57*–*60*).

## MATERIALS AND METHODS

### Sample fabrication and structural characterization

#### Materials

Lead (II) iodide (99.999%) and γ-butyrolactone (≥99%) were obtained from Sigma-Aldrich; phenethylammonium iodide was obtained from Greatcell Solar. All chemicals were used as received.

#### Synthesis

The perovskite thin films were fabricated by a two-step spin-coating method in ambient conditions. Specifically, the perovskite precursor solution was prepared by dissolving 0.9 mmol of lead (II) iodide and 1.8 mmol of phenethylammonium iodide into 0.54 ml of γ-butyrolactone. The substrates were treated with air plasma for 10 min to ensure good solution wettability. The perovskite films were fabricated by dropping 50 μl of precursor solution onto the substrates and going through a two-step spin-coating program with 1000 rpm for 5 s and 3000 rpm for 45 s. Subsequently, the film was annealed at 80°C on a hot plate for 15 min.

#### Structural characterization

The crystalline orientations of the perovskite films on various substrates were measured by XRD using θ-2θ scans with a Rigaku SmartLab x-ray diffractometer. The film thickness, morphology, and interfacial quality of multilayer stacks were examined by SEM (Hitachi SU8230).

### NC synthesis

#### Materials

Cadmium acetylacetonate (99.9%), indium fluoride (99.9%), indium acetate (99.99%), octadecene (90%), and oleic acid (90%) were obtained from Sigma-Aldrich and used as received. Isopropanol and toluene for washing the particles were American Chemical Society grade from Thermo Fisher Scientific.

#### Synthesis

Colloidal NCs of doped cadmium oxide were prepared following literature procedures with small modifications (*51*, *61*, *62*). All reactions consisted of 25 ml of octadecene, 1 ml of oleic acid, and 1 mmol of total metal precursors; the precursors were loaded into a three-neck, 50-ml reaction flask and heated to 120°C and held for 1 hour under vacuum (~1 torr), then heated rapidly under nitrogen atmosphere to the boiling point (~316°C). The reaction was held at the boiling point of the solution for times between 5 and 40 min before the reaction medium turned from colorless to brown or green. After this color change, the reaction was maintained at 316°C for an additional 10 min before being cooled back to room temperature by removal of a heating mantle. At room temperature, the reaction was decanted into two centrifuge tubes, and 10 ml of toluene was added. Then, isopropanol was added as an antisolvent until flocculation of the particles occurred. The samples were centrifuged for 5 min at 4000 rpm. Then, the pallet was redispersed in 5 ml of toluene. The solution was flocculated again with additional isopropanol and centrifuged for 5 min at 4000 rpm. The resulting pellet was redispersed in 3 ml of toluene and centrifuged at 2000 rpm for 2 min. The supernatant was saved. After 1 week, the solution was filtered through a 0.45-μm Teflon filter to remove precipitates. To change the frequency of the LSPR, the type and amount of doping were controlled through the input metal precursors. Cadmium acetylacetonate was used as the cadmium precursor for 80 to 99% of the total metal content. Indium doping was achieved using between 1 and 20% indium acetate. Indium and fluorine codoping was achieved by adding between 8 and 20% indium fluoride. The indium acetate doping concentrations in ICO-1 and ICO-2 were 20 and 5%, respectively. The indium fluoride doping concentration of FICO was 15%. The micrographs of the NCs were acquired by a JEOL 2100F transmission electron microscope operated at 200 kV with a ×60,000 magnification.

### Fabrication of membrane structure

#### Silicon etching

KOH solution (19.2 ml of 45 wt %) was added to 11.4 ml of H_2_O to reach a 32–wt % concentration. Then, 3 ml of IPA was added to the 32–wt % KOH solution to minimize the surface roughness of the etched samples. The solution was heated to 65°C, and SiN*_x_*/Si wafers (i.e., 600-μm-thick Si coated with 265 nm of low-stress SiN*_x_* on both sides) were etched for 24 hours. Before dipping the SiN*_x_*/Si wafer into the etch solution, a pinhole was created on its backside using a diamond scribe for enabling contact between Si and the KOH etcher.

#### Fabrication of the membrane samples

PEA precursor solution was prepared by dissolving 0.75 mmol of lead (II) iodide and 1.5 mmol of PEAI into 0.5 ml of *N*,*N*′-dimethylformamide. The etched SiN*_x_*/Si wafers were treated with air plasma for 10 min to ensure good solution wettability. PEA films were fabricated by spin-coating 50 μl of the as-prepared precursor solution onto preheated (150°C) SiN*_x_*/Si at 3000 rpm for 30 s with an acceleration of 1000 rpm/s. Then, 50 μl of ICO NC solution was spin-coated onto the substrates at 1000 rpm for 30 s with an acceleration of 500 rpm/s: (i) For the PEA/SiN*_x_*/ICO membrane structure, the ICO NC solution was first spin-coated on the back side (i.e., the etched side) of the SiN*_x_*/Si substrate, then PEA solution was spin-coated on the front side; (ii) for the ICO/PEA/SiN*_x_* membrane structure, the ICO NC solution was spin-coated onto the PEA film, with the latter first deposited on the SiN*_x_*/Si substrate. A schematic illustration for the fabrication of these two structures is shown in fig. S12.

### Finite-element simulations of heat transfer

The Heat Transfer module available in COMSOL Multiphysics 5.3a was used to simulate the transient thermal transport process along the cross-plane direction for comparison with experimental data. The calculation was performed in the time domain by solving the equation of heat conduction (i.e., Fourier’s law), $\rho C_{P}\frac{\partial T}{\partial t}+\nabla \cdot \left(-\kappa \nabla T\right)=0$, where ρ is the mass density, $C_{P}$ is the specific heat capacity, κ is the thermal conductivity, and T is the lattice temperature.

The heat capacity and thermal conductivity for silicon wafer were taken to be 710 J kg^−1^ K^−1^ and 142.2 W m^−1^ K^−1^, respectively. The thermal conductivity and heat capacity of PET were taken to be 0.19 W m^−1^ K^−1^ and 1000 J kg^−1^ K^−1^, respectively (Fire Safety Research Institute; https://materials.fsri.org/materialdetail/polyethylene-terephthalate-pet). The density, heat capacity, and thermal conductivity for cadmium oxide were taken from literature as 8101.4 kg m^−3^, 43.1 J mol^−1^ K^−1^ (or equivalently 335.6 J kg^−1^ K^−1^), and 5.6 to 9.3 W m^−1^ K^−1^, respectively (*63*, *64*). The heat capacity of the TCO films was calculated by using a volume filling ratio of 41% for the inorganic core, 23% for the organic ligands, and 36% for the empty space between the particles. Such volume filling ratios were estimated by assuming that the inorganic core has a spherical shape with a diameter of 9.5 nm, the oleic ligands have a length of 1.5 nm, and the particles are randomly close-packed (i.e., empty space between the particles occupies a volume fraction of 36%). The heat capacity (*65*) and mass density for oleic acid at 298 K were taken to be 1950 J kg^−1^ K^−1^ and 895 kg m^−3^, respectively. The heat capacity and mass density of the TCO film were determined to be 429.8 J kg^−1^ K^−1^ and 3527.4 kg m^−3^, respectively (by weighted sums of the inorganic and organic components comprising the film). The heat capacity for PEA was taken from the literature (*35*).

When simulating the ICO/PEA/substrate stack, a uniform temperature rise of 10 K at time zero was assumed for the ICO film, whereas the initial temperature rise in PEA was taken to be zero, because the MIR absorption of ICO is about an order of magnitude higher than PEA. When simulating the PEA/substrate stack (i.e., no ICO), the initial temperature in PEA was peaked at the surface and decayed exponentially along the depth following the Beer-Lambert law. The absorption coefficient of the MIR pump in the PEA film was determined to be 6140 cm^−1^ using the data shown in Fig. 2C. In accordance with the penetration depth of the probe at ~500 nm (~140 nm; fig. S1), the temperature of the top 140-nm thickness of the PEA film from the simulations was averaged, the normalized temporal decay of which was compared with the normalized, experimental ∆R/R at ~500 nm. It is to be noted that in the small temperature perturbation regime of the TR experiments (as indicated by the fluence-independent decay kinetics; fig. S15), the ∆R/R scales linearly with the lattice temperature, justifying the direct comparison between the simulated temperature decay and the measured kinetics of ∆R/R.

### Steady-state optical characterization

The steady-state optical reflectance experiments were performed using a customized micro–reflectance spectroscopy setup as reported previously (*38*), with a slight modification that the light source was replaced by a supercontinuum laser (DISCO-2-UV, Leukos). A liquid-nitrogen cryostat (VPF-100, Lake Shore) was used to vary the sample’s temperature from 78 to 300 K at a vacuum level better than 1 × 10^−4^ torr. The MIR transmittance spectra were measured by Fourier-transform infrared spectroscopy (Nicolet 6700 FT-IR) with a mercury-cadmium-telluride detector.

### IPVP TR experiments

The IPVP TR measurements with a nanosecond-to-millisecond time window were based on a setup reported previously (*44*, *66*). Briefly, the idler output from a high-energy MIR optical parametric amplifier (OPA; Orpheus-ONE-HE, Light Conversion) served as the pump. The OPA was powered by a 170-fs Yb:KGW laser amplifier (Pharos, Light Conversion) with 0.9-mJ input energy operated at a 2-kHz repetition rate and reduced into 1 kHz by an optical chopper. The broadband probe pulses at a 2-kHz repetition rate were produced by a supercontinuum laser (DISCO-2-UV, Leukos), which was electronically triggered and delayed from the femtosecond pump laser with a digital delay generator (DG645, Stanford Research Systems). The pump pulse was focused onto the sample using a CaF_2_ lens of 200-mm focal length, which yielded a spot size of ~220 μm in diameter. The probe pulse was focused onto the sample using a 100-mm focal-length, achromatic doublet lens, providing a spot size of ~50 μm in diameter. The reflected probe pulses were captured and processed with a universal serial bus–coupled complementary metal-oxide semiconductor spectrometer (AvaSpec-ULS2048CL-EVO, Avantes).

### All-optical MIR detection measurements

MIR supercontinuum lasers (SC4500 from Thorlabs or Electro MIR 4.8 from Leukos) were used as the MIR light sources, which offer broadband output from 2.2 to 4.8 μm. The repetition rate of 50 MHz (for SC4500) or 250 kHz (for Electro MIR 4.8) is much higher than the chopping rate of less than 5 kHz. Compared to the thermal response time of the sample (i.e., tens of microseconds; Fig. 1F) and the optical chopper frequency (100 to 5000 Hz), the high–repetition rate MIR lasers can be treated as continuous-wave sources. Several MIR band-pass filters (FB1900-200, FB3250-500, FB3330-150, FB3750-500, and FB4000-500, all from Thorlabs) were used to select the wavelength of the MIR light incident on the sample. The measured transmittance spectra of the MIR filters are plotted in fig. S9. An optical chopper was used to turn the MIR beam on and off at a frequency of 100 Hz for Fig. 3 (unless otherwise specified) or 80 Hz for Figs. 4 and 5. A CaF_2_ lens was used to focus the MIR laser onto the sample with a spot size of 680 μm in diameter. For MIR measurement at 10.6 μm, we used a continuous-wave CO_2_ gas laser, which was focused on the sample using a ZnSe lens. A combination of two ZnSe MIR linear polarizers was used to vary the power of the MIR laser incident on the sample.

For experiments shown in Fig. 3, the samples were probed by a high-power green light-emitting diode (LED) source (SOLIS-505C, Thorlabs). The output from the LED was wavelength filtered by a 500-nm band-pass filter (10BPF10-500, Newport) or a 510-nm band-pass filter (FBH510-10, Thorlabs). For experiments shown in Figs. 4 and 5, the samples were probed by a supercontinuum laser coupled with a monochromator (Iceblink and Boreal, FYLA Inc.). The probe spectral width is ~8 nm in its full width at half maximum. The probe light reflected by the sample was directed into a PMT (H10722-20, Hamamatsu), whose voltage output was measured by a lock-in amplifier (SR860, Stanford Research Systems) with the optical chopper providing the reference signal. The control voltage of the PMT was adjusted to yield a constant output voltage of 0.5 V in the measurements for Fig. 3, or 0.1 V in the measurements for Figs. 4 and 5, to enable comparison between different samples.

## Supplementary Materials

### This PDF file includes:

Figs. S1 to S16 Table S1 References
