# Supplementary material for: Mid-infrared photodetection with 2D metal halide perovskites at ambient temperature
Source: Sci Adv. 2024 Dec 13;10(50):eadk2778. doi: 10.1126/sciadv.adk2778 (PMC11641002; doi:10.1126/sciadv.adk2778)
Supplement: Supplementary file 1 — Figs. S1 to S16 Table S1 References [file sciadv.adk2778_sm.pdf]

Science Advances
AAAS

Supplementary Materials for

Mid-infrared photodetection with 2D metal halide perovskites at ambient temperature

Yanyan Li *et al.*

Corresponding author: Peijun Guo, peijun.guo@yale.edu

Sci. Adv. **10**, eadk2778 (2024)
DOI: 10.1126/sciadv.adk2778

This PDF file includes:

Figs. S1 to S16
Table S1
References

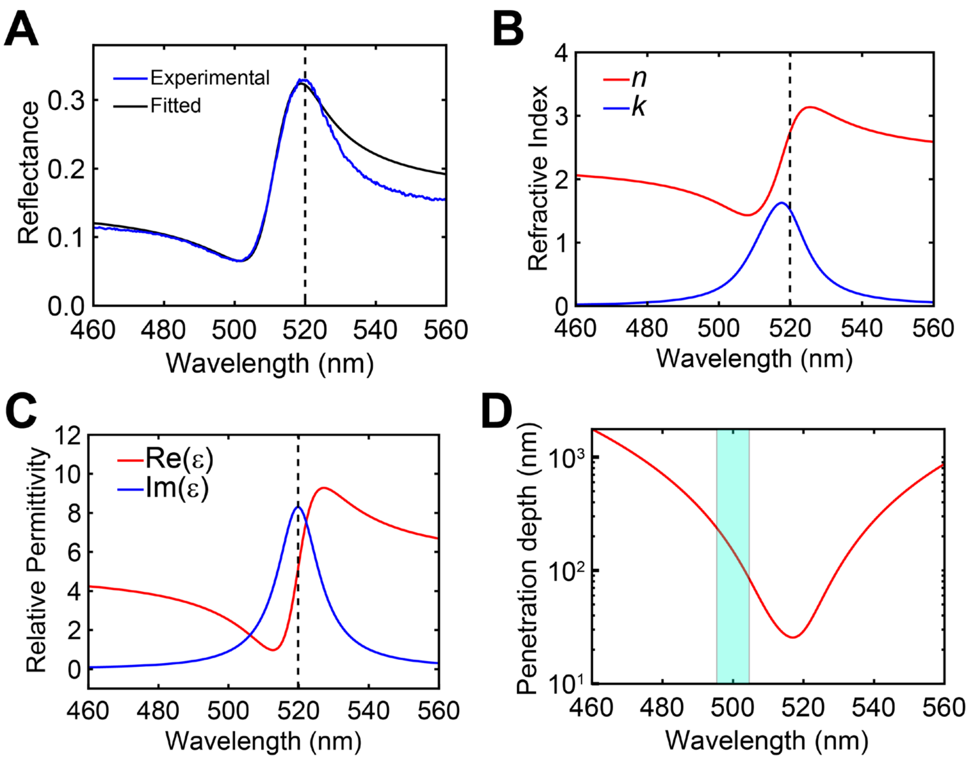

Figure S1. Fitting of the reflectance of PEA single crystal using the Lorentzian model. (**A**) Blue: reflectance spectrum of single-crystal PEA at room temperature. Black: calculated reflectance spectrum of PEA with the relative permittivity described by the Lorentzian oscillator $\varepsilon(\omega) = \varepsilon_\infty + \frac{{A_\mathrm{L}}^2}{{\omega_\mathrm{L}}^2 - \omega^2 - i\omega\gamma}$, where $\varepsilon_\infty = 5.07$ is the high-frequency permittivity, $\omega_\mathrm{L} = 2.39$ eV is the exciton energy, $\gamma = 67$ meV is the exciton damping factor, and $A_\mathrm{L} = 1.15$ eV is the oscillator strength. (**B**) Complex refractive index and (**C**) Complex relative permittivity of PEA calculated from the Lorentzian oscillator model. (**D**) Optical penetration depth, which is calculated as $1/\alpha$; α is the absorption coefficient determined as $\alpha = \frac{2\pi\varepsilon''}{n\lambda}$ (ε'' is the imaginary part of relative permittivity, n is the real part of refractive index, and λ is the wavelength). The optical penetration depth is equivalent to the depth at which the light intensity drops to $1/\mathrm{e}$ of the original intensity.

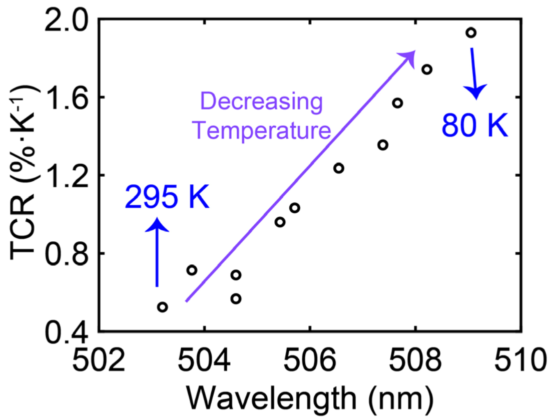

Figure S2. Temperature coefficient of reflectivity of PEA single crystal versus temperature. The *x*-axis represents the temperature-dependent wavelength at which single-crystal PEA exhibits the largest TCR. The *y*-axis shows the TCR values at the corresponding wavelengths.

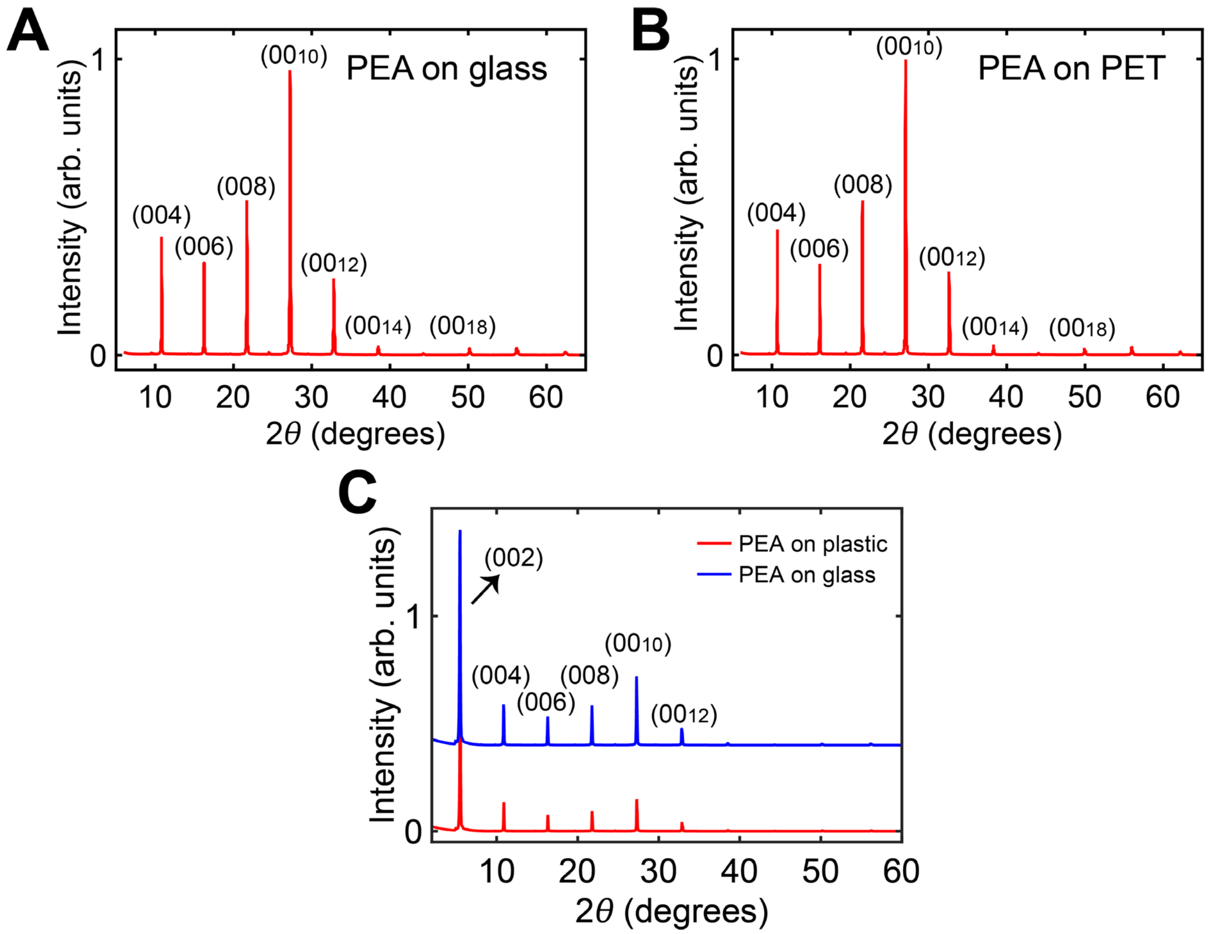

Figure S3. Structural characterization of PEA thin films. X-ray diffraction pattern of spin-coated PEA films on a glass substrate in (**A**) and on a PET substrate in (**B**). Data shown in (**C**) include the (002) peak between 2 to 10 degrees (the two curves are offset for clarity).

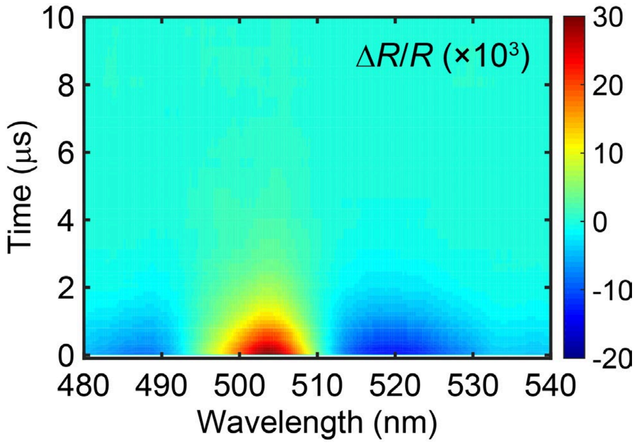

Figure S4. Transient spectral map of $\Delta R/R$ for 640 nm-thick spin-coated PEA film on Si measured at room temperature. The pump wavelength was 3170 nm and the pump fluence was 3.6 mJ·cm^{-2}.

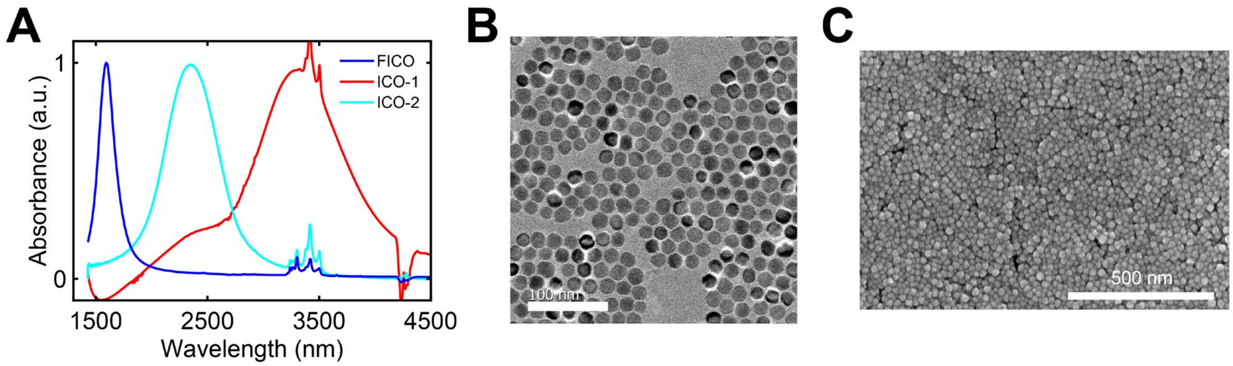

Figure S5. Optical and morphological characterization of the ICO nanocrystals. (**A**) Infrared absorbance spectra of the three types of ICO nanocrystals dispersed in toluene. (**B**) Transmission electron microscopy image of ICO-1 nanocrystals taken at 60,000X magnification, showing the uniform size distribution of the nanocrystals. (**C**) Scanning electron microscopy image showing the morphology of spin-coated ICO-1 nanocrystals on PEA/PET.

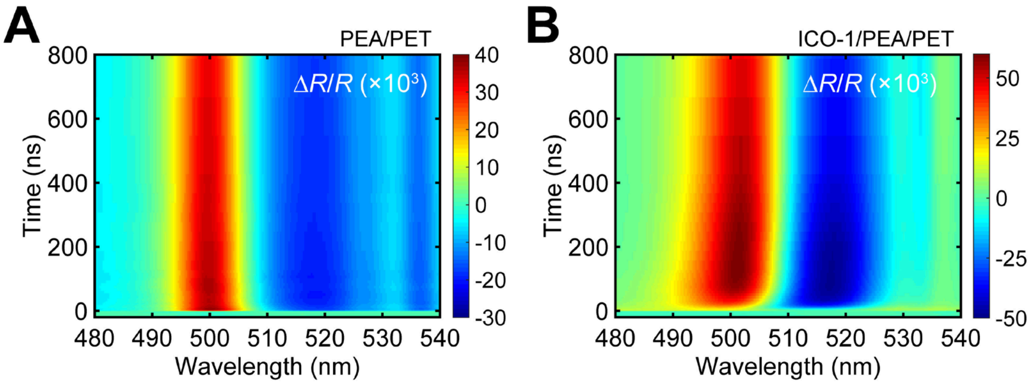

Figure S6. Transient optical response of PEA film without and with ICO nanocrystal coating. (**A**) Transient $\Delta R/R$ spectral map for a 940 nm-thick PEA film on PET measured at room temperature (pump wavelength: 3170 nm; fluence: 3.6 mJ·cm^{-2}). (**B**) Transient $\Delta R/R$ spectral map of ICO-1/PEA/PET at room temperature (pump wavelength: 3170 nm; fluence: 3.9 mJ·cm^{-2}).

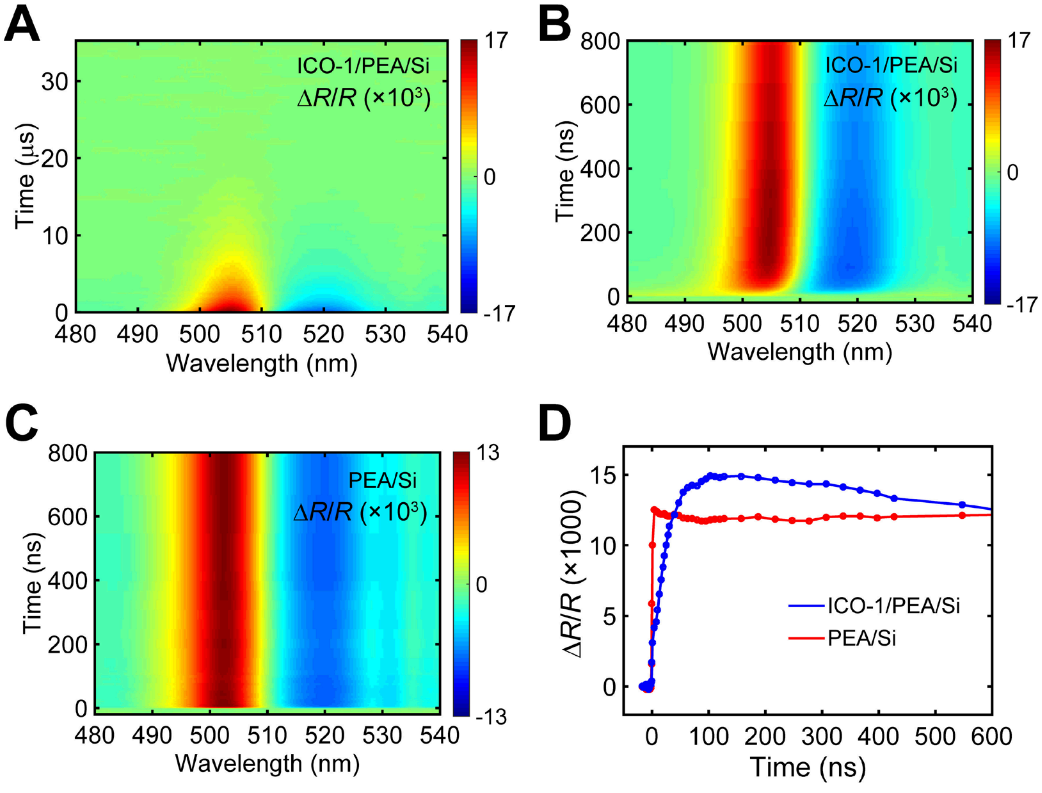

Figure S7. Transient optical response of PEA on silicon. (**A**) and (**B**) Transient $\Delta R/R$ spectral maps for ICO-1/PEA/Si measured at room temperature. (**C**) Transient $\Delta R/R$ spectral maps for PEA/Si measured at room temperature. (**D**) Comparison of the $\Delta R/R$ kinetics of the ICO-1/PEA/Si and the PEA/Si samples at 502 nm.

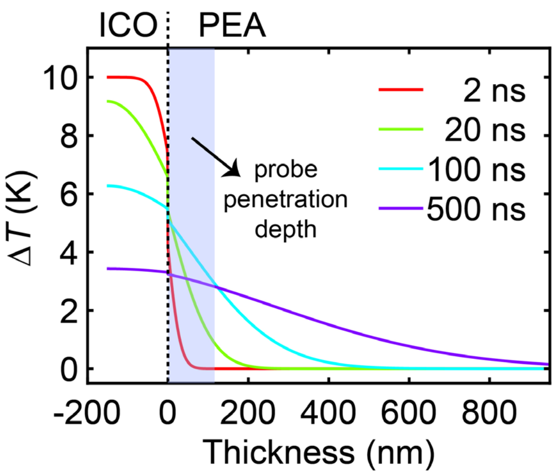

Figure S8. Simulated temperature profiles of ICO/PEA/PET. Simulated temperature profiles of an ICO/PEA/PET stack at various delay times following impulsive thermal excitation of the ICO film at time zero, showing energy transfer from ICO to PEA.

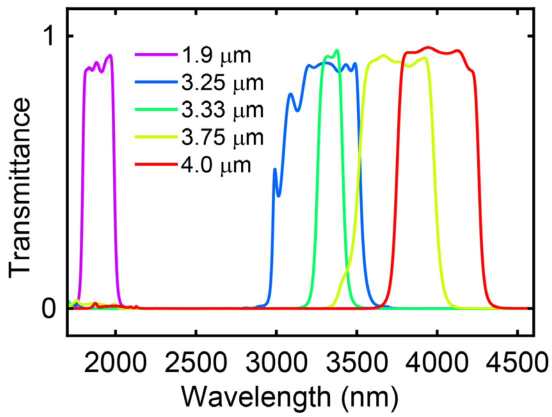

Figure S9. Transmittance spectra of the five MIR bandpass filters used in the MIR detection experiments. The transmittance curves are for FB1900-200, FB3250-500, FB3330-150, FB3750-500, and FB4000-500 filters (from short to long wavelength) measured with FTIR.

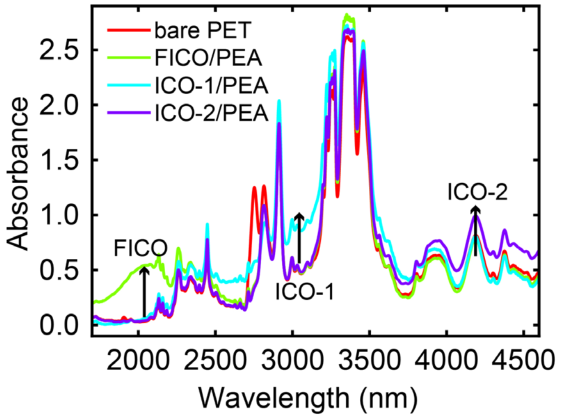

Figure S10. Infrared absorbance spectra for a bare PET substrate as well as PET substrates coated with PEA/Al_2O_3 and terminated with thin films of the three types of ICO nanocrystals. The enhanced absorption from the nanocrystal films is indicated by the black arrows in the figure. Note that the absorbance of MIR light by the PET substrate cannot be used to fulfill MIR photodetection function, because the absorption coefficient of MIR light for PET (0.5 mm thick) is much weaker than that for the ICO nanocrystals (<200 nm thick); only the ICO nanocrystal film can enable a heat concentrating effect for the PEA film.

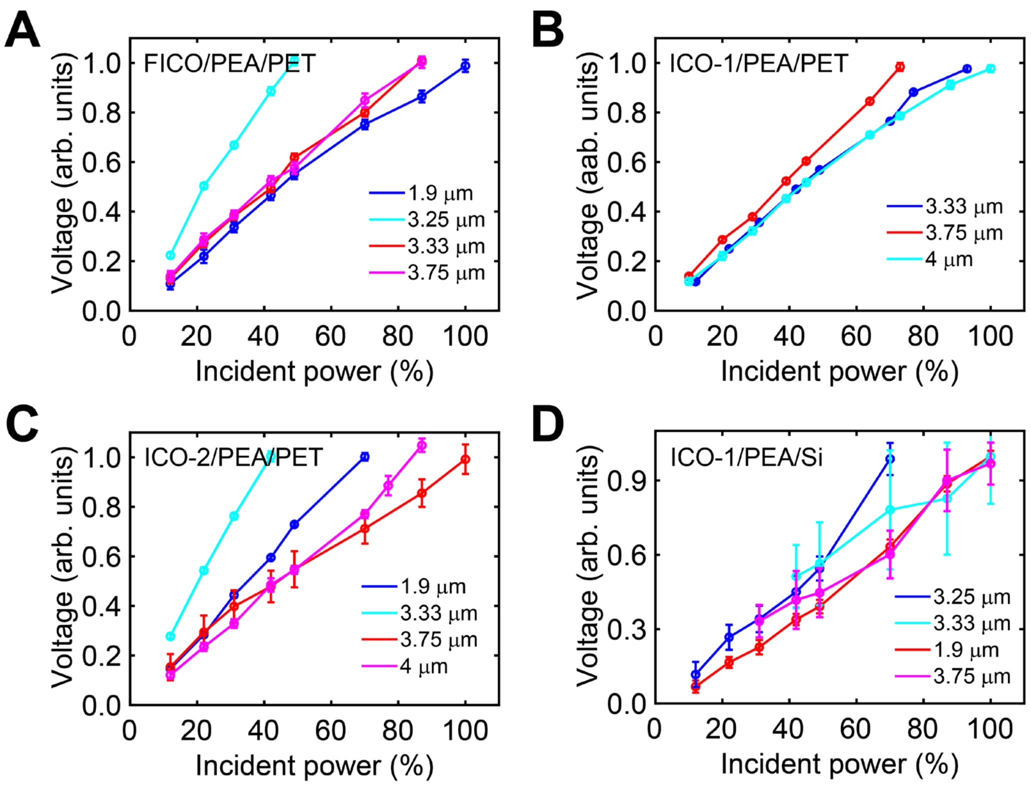

Figure S11. Tests of linear responsivity to the MIR power for the samples. Lock-in voltages measured as functions of the incident power of the MIR source used in the all-optical MIR detection experiments. Data for samples employing different ICO NCs are shown: FICO in (**A**), ICO-1 in (**B**), ICO-2 in (**C**), and ICO-1 with Si substrate in (**D**). Note that **1)** the magnitudes of MIR powers for different wavelengths are separately normalized, and **2)** the voltages measured with Si substrate in (**D**) have lower signal-to-noise ratios.

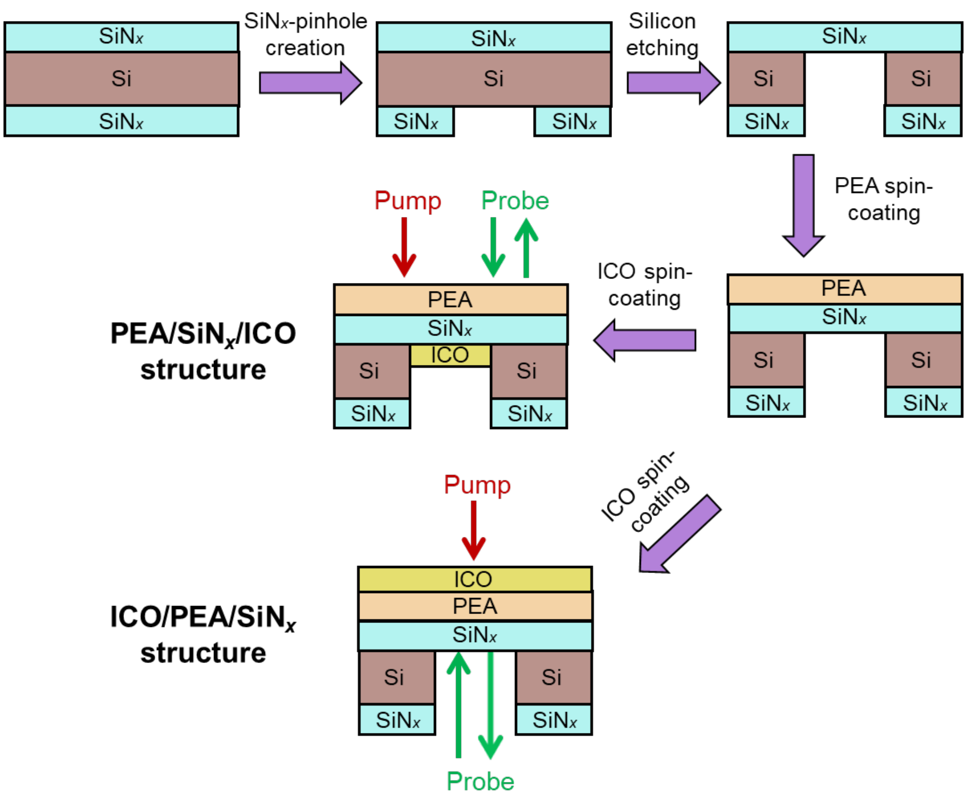

Figure S12. Schematic illustration for the fabrication procedure for the two types of membrane-based structures. Both the PEA/SiN_x/ICO used in Fig. 4 and ICO/PEA/SiN_x used in Fig. 5 are shown.

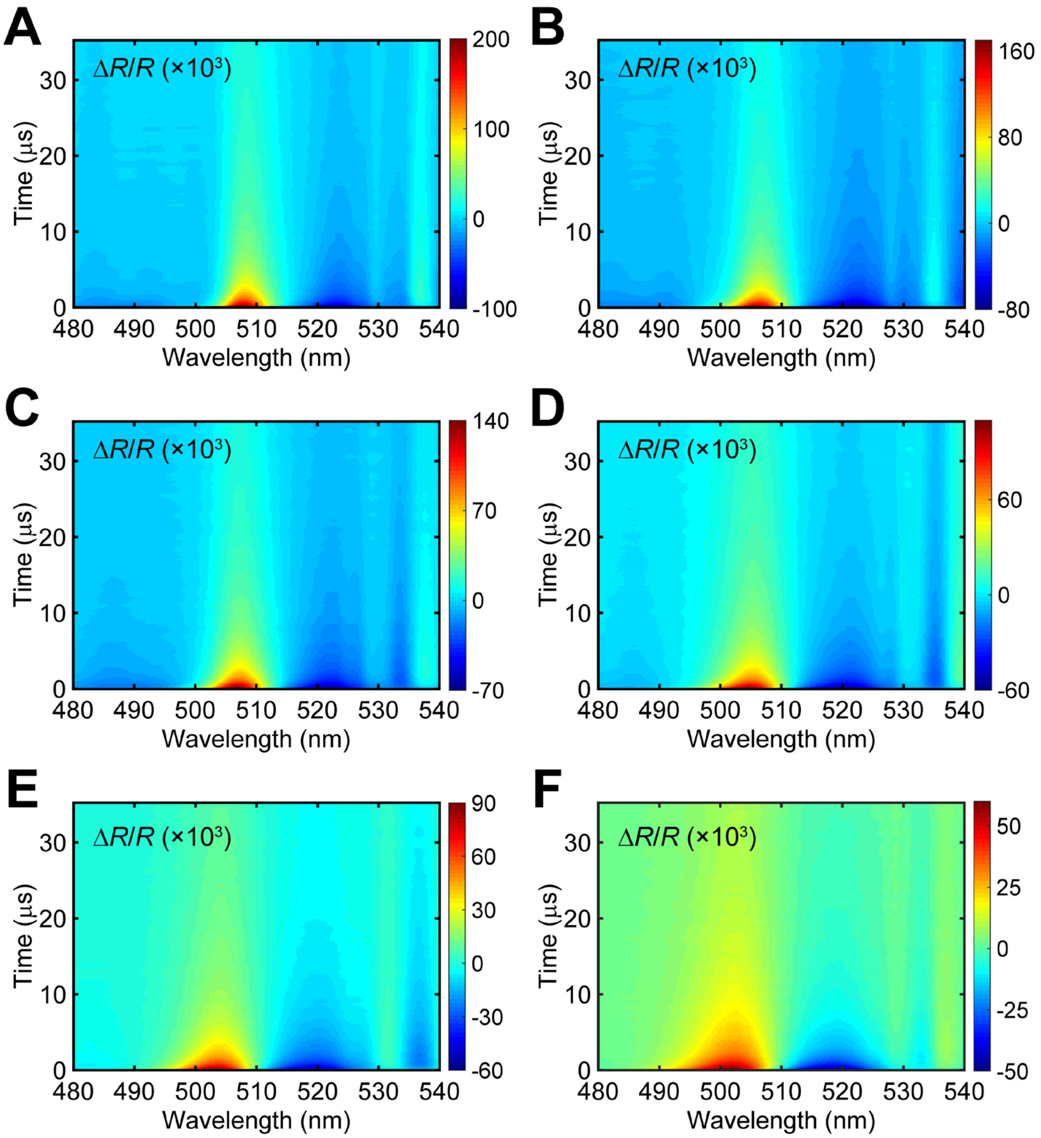

Figure S13. Temperature-dependent $\Delta R/R$ spectral maps. (**A**) to (**F**) Transient $\Delta R/R$ spectral maps for a single-crystal PEA measured at 80 K, 130 K, 170 K, 210 K, 250 K and 290 K, respectively (pump wavelength: 3170 nm; fluence: 2.3 mJ·cm^{-2}).

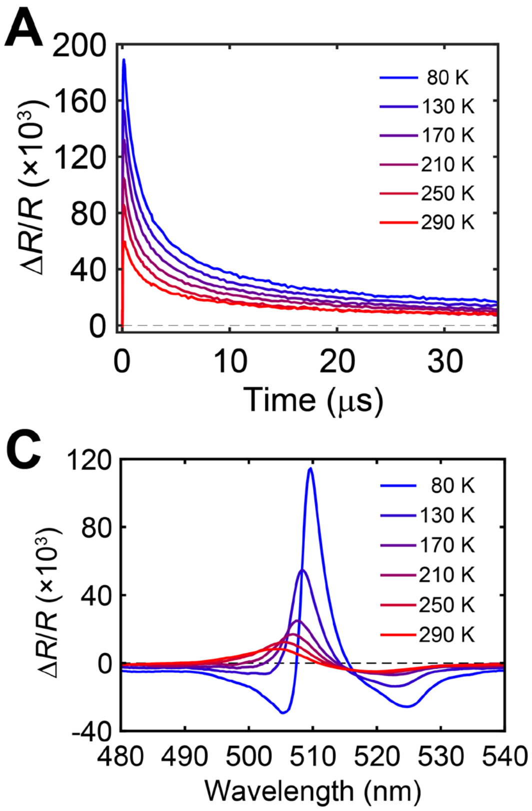

Figure S14. Temperature-dependent transient reflection measurements. (**A**) Temperature dependence of the $\Delta R/R$ kinetics measured for PEA/PET (thickness of PEA is 940 nm). The wavelength used in the plots is chosen to maximize the $\Delta R/R$ at each temperature. (**B**) Normalized plots of the data shown in (**A**). (**C**) Temperature-dependent $\Delta R/R$ spectra at 10 ns delay time for a PEA single crystal pumped at 3170 nm at fluence of 2.2 mJ$\cdot$cm^{-2}.

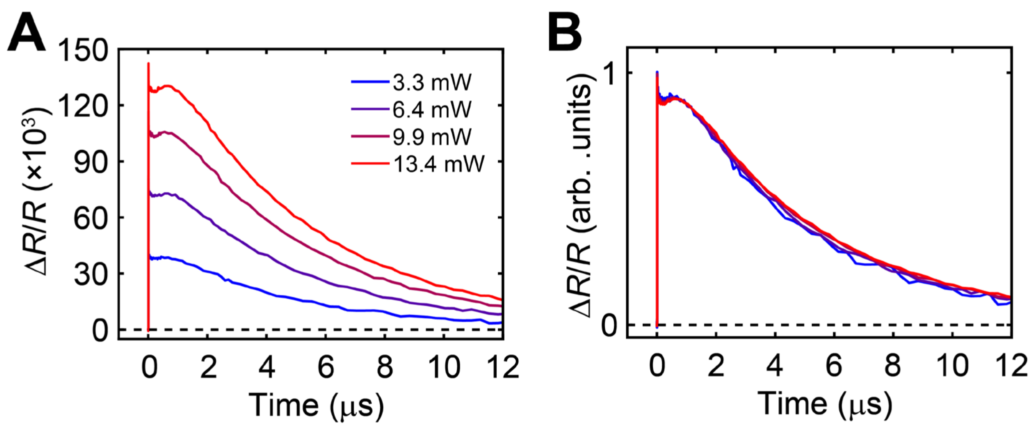

Figure S15. Pump power-dependent transient reflection measurements. (**A**) Pump fluence-dependent $\Delta R/R$ for a 640 nm-thick PEA on Si substrate measured at room temperature with (**A**) pump wavelength of 3170 nm. (**B**) Normalized version of the curves shown in (**A**).

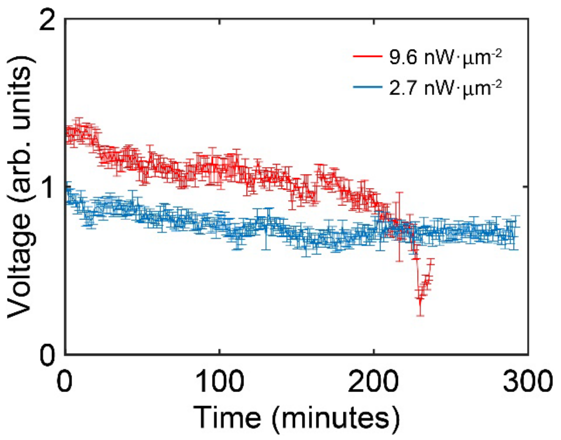

Figure S16. Stability tests. Stability test of SiN_x membrane-supported PEA film with its back side covered by a thin layer (<100 nm) of dielectric. The power densities indicated in the legend are calculated using the absorbed MIR powers by the sample, not the powers of incident MIR light.

**Devices**	**Materials**	**Operation wavelength**	**Sensitivity**	**Temperature**	**Ref.**
All-optical detection	$(PEA)_2PbI_4$	2-4.5 μm<br>10.6 μm	< 10 pW·μm^{-2}<br>< 20 pW·μm^{-2}	Room temperature (RT)	This work
Nanoparticle-on-resonator	Au antenna and Biphenyl-4-thiol (BPT)	8.5-12.6 μm	1 μW·μm^{-2}	RT	[11]
Nano transducers	$NaYF_4$:Nd^{3+}@$NaYF_4$	4-11 μm	300 pW·μm^{-2}	RT	[12]
Charge density wave compound-based device	1T-TaS_2	visible to terahertz	(300 pW Hz$^{-1/2}$)	RT	[13]
Bolometer	Graphene	1.5 μm	(10 pW Hz$^{-1/2}$)	5 K	[67]
CMOS THz camera and polarimeter	CdSe/CdS/ZnS nanocrystals	0.1–1.5 THz	0.13 pW·μm^{-2} (**picosecond pulses** only)	RT	[68]
Microbolometer	Silicon nanomembrane	12.2 μm	115 nW·μm^{-2}	RT	[69]

Table S1. Comparison of different types of emerging schemes for long-wavelength light detection taken from various references.

REFERENCES AND NOTES

1. A. V. Muraviev, V. O. Smolski, Z. E. Loparo, K. L. Vodopyanov, Massively parallel sensing of trace molecules and their isotopologues with broadband subharmonic mid-infrared frequency combs. *Nat. Photonics* **12**, 209–214 (2018).

2. H. Lin, L. Li, Y. Zou, S. Danto, J. D. Musgraves, K. Richardson, S. Kozacik, M. Murakowski, D. Prather, P. T. Lin, V. Singh, A. Agarwal, L. C. Kimerling, J. Hu, Demonstration of high-Q mid-infrared chalcogenide glass-on-silicon resonators. *Opt. Lett.* **38**, 1470–1472 (2013).

3. C. R. Petersen, N. Prtljaga, M. Farries, J. Ward, B. Napier, G. R. Lloyd, J. Nallala, N. Stone, O. Bang, Mid-infrared multispectral tissue imaging using a chalcogenide fiber supercontinuum source. *Opt. Lett.* **43**, 999–1002 (2018).

4. S. P. Garaba, J. Aitken, B. Slat, H. M. Dierssen, L. Lebreton, O. Zielinski, J. Reisser, Sensing ocean plastics with an airborne hyperspectral shortwave infrared imager. *Environ. Sci. Technol.* **52**, 11699–11707 (2018).

5. K. Zou, K. Pang, H. Song, J. Fan, Z. Zhao, H. Song, R. Zhang, H. Zhou, A. Minoofar, C. Liu, X. Su, N. Hu, A. McClung, M. Torfeh, A. Arbabi, M. Tur, A. E. Willner, High-capacity free-space optical communications using wavelength- and mode-division-multiplexing in the mid-infrared region. *Nat. Commun.* **13**, 7662 (2022).

6. S. McSherry, M. Webb, J. Kaufman, Z. Deng, A. Davoodabadi, T. Ma, E. Kioupakis, K. Esfarjani, J. T. Heron, A. Lenert, Nanophotonic control of thermal emission under extreme temperatures in air. *Nat. Nanotechnol.* **17**, 1104–1110 (2022).

7. R. K. Bhan, V. Dhar, Recent infrared detector technologies, applications, trends and development of HgCdTe based cooled infrared focal plane arrays and their characterization. *Opto-Electron. Rev.* **27**, 174–193 (2019).

8. A. Rogalski, HgCdTe infrared detector material: History, status and outlook. *Rep. Prog. Phys.* **68**, 2267–2336 (2005).

9. P. V. K. Yadav, I. Yadav, B. Ajitha, A. Rajasekar, S. Gupta, Y. Ashok Kumar Reddy, Advancements of uncooled infrared microbolometer materials: A review. *Sens. Actuators A Phys.* **342**, 113611 (2022).

10. H. Wang, X. Yi, G. Huang, J. Xiao, X. Li, S. Chen, IR microbolometer with self-supporting structure operating at room temperature. *Infrared Phys. Technol.* **45**, 53–57 (2004).

11. A. Xomalis, X. Zheng, R. Chikkaraddy, Z. Koczor-Benda, E. Miele, E. Rosta, G. A. E. Vandenbosch, A. Martínez, J. J. Baumberg, Detecting mid-infrared light by molecular frequency upconversion in dual-wavelength nanoantennas. *Science* **374**, 1268–1271 (2021).

12. L. Liang, C. Wang, J. Chen, Q. J. Wang, X. Liu, Incoherent broadband mid-infrared detection with lanthanide nanotransducers. *Nat. Photonics* **16**, 712–717 (2022).

13. D. Wu, Y. Ma, Y. Niu, Q. Liu, T. Dong, S. Zhang, J. Niu, H. Zhou, J. Wei, Y. Wang, Z. Zhao, N. Wang, Ultrabroadband photosensitivity from visible to terahertz at room temperature. *Sci. Adv.* **4**, eaao3057 (2018).

14. S. Castilla, I. Vangelidis, V.-V. Pusapati, J. Goldstein, M. Autore, T. Slipchenko, K. Rajendran, S. Kim, K. Watanabe, T. Taniguchi, L. Martín-Moreno, D. Englund, K.-J. Tielrooij, R. Hillenbrand, E. Lidorikis, F. H. L. Koppens, Plasmonic antenna coupling to hyperbolic phonon-polaritons for sensitive and fast mid-infrared photodetection with graphene. *Nat. Commun.* **11**, 4872 (2020).

15. L. Zeng, D. Wu, J. Jie, X. Ren, X. Hu, S. P. Lau, Y. Chai, Y. H. Tsang, Van der Waals epitaxial growth of mosaic-like 2D platinum ditelluride layers for room-temperature mid-infrared photodetection up to 10.6 μm. *Adv. Mater.* **32**, 2004412 (2020).

16. A. Kojima, K. Teshima, Y. Shirai, T. Miyasaka, Organometal halide perovskites as visible-light sensitizers for photovoltaic cells. *J. Am. Chem. Soc.* **131**, 6050–6051 (2009).

17. S. Bai, P. Da, C. Li, Z. Wang, Z. Yuan, F. Fu, M. Kawecki, X. Liu, N. Sakai, J. T.-W. Wang, S. Huettner, S. Buecheler, M. Fahlman, F. Gao, H. J. Snaith, Planar perovskite solar cells with long-term stability using ionic liquid additives. *Nature* **571**, 245–250 (2019).

18. H. Wei, Y. Fang, P. Mulligan, W. Chuirazzi, H.-H. Fang, C. Wang, B. R. Ecker, Y. Gao, M. A. Loi, L. Cao, J. Huang, Sensitive X-ray detectors made of methylammonium lead tribromide perovskite single crystals. *Nat. Photonics* **10**, 333–339 (2016).

19. B. R. Sutherland, E. H. Sargent, Perovskite photonic sources. *Nat. Photonics* **10**, 295–302 (2016).

20. S. Tan, T. Huang, I. Yavuz, R. Wang, T. W. Yoon, M. Xu, Q. Xing, K. Park, D.-K. Lee, C.-H. Chen, R. Zheng, T. Yoon, Y. Zhao, H.-C. Wang, D. Meng, J. Xue, Y. J. Song, X. Pan, N.-G. Park, J.-W. Lee, Y. Yang, Stability-limiting heterointerfaces of perovskite photovoltaics. *Nature* **605**, 268–273 (2022).

21. Z. Xiao, R. A. Kerner, L. Zhao, N. L. Tran, K. M. Lee, T.-W. Koh, G. D. Scholes, B. P. Rand, Efficient perovskite light-emitting diodes featuring nanometre-sized crystallites. *Nat. Photonics* **11**, 108–115 (2017).

22. J.-P. Correa-Baena, Y. Luo, T. M. Brenner, J. Snaider, S. Sun, X. Li, M. A. Jensen, N. T. P. Hartono, L. Nienhaus, S. Wieghold, J. R. Poindexter, S. Wang, Y. S. Meng, T. Wang, B. Lai, M. V. Holt, Z. Cai, M. G. Bawendi, L. Huang, T. Buonassisi, D. P. Fenning, Homogenized halides and alkali cation segregation in alloyed organic-inorganic perovskites. *Science* **363**, 627–631 (2019).

23. A. D. Wright, C. Verdi, R. L. Milot, G. E. Eperon, M. A. Pérez-Osorio, H. J. Snaith, F. Giustino, M. B. Johnston, L. M. Herz, Electron–phonon coupling in hybrid lead halide perovskites. *Nat. Commun.* **7**, 11755 (2016).

24. Y. Guo, O. Yaffe, T. D. Hull, J. S. Owen, D. R. Reichman, L. E. Brus, Dynamic emission Stokes shift and liquid-like dielectric solvation of band edge carriers in lead-halide perovskites. *Nat. Commun.* **10**, 1175 (2019).

25. X. Gong, O. Voznyy, A. Jain, W. Liu, R. Sabatini, Z. Piontkowski, G. Walters, G. Bappi, S. Nokhrin, O. Bushuyev, M. Yuan, R. Comin, D. McCamant, S. O. Kelley, E. H. Sargent, Electron–phonon interaction in efficient perovskite blue emitters. *Nat. Mater.* **17**, 550–556 (2018).

26. K. Miyata, D. Meggiolaro, M. T. Trinh, P. P. Joshi, E. Mosconi, S. C. Jones, F. De Angelis, X.-Y. Zhu, Large polarons in lead halide perovskites. *Sci. Adv.* **3**, e1701217 (2017).

27. B. Guzelturk, T. Winkler, T. W. J. Van de Goor, M. D. Smith, S. A. Bourelle, S. Feldmann, M. Trigo, S. W. Teitelbaum, H.-G. Steinrück, G. A. de la Pena, R. Alonso-Mori, D. Zhu, T. Sato, H. I. Karunadasa, M. F. Toney, F. Deschler, A. M. Lindenberg, Visualization of dynamic polaronic strain fields in hybrid lead halide perovskites. *Nat. Mater.* **20**, 618–623 (2021).

28. B. Saparov, D. B. Mitzi, Organic–inorganic perovskites: Structural versatility for functional materials design. *Chem. Rev.* **116**, 4558–4596 (2016).

29. X. Li, J. M. Hoffman, M. G. Kanatzidis, The 2D halide perovskite rulebook: How the spacer influences everything from the structure to optoelectronic device efficiency. *Chem. Rev.* **121**, 2230–2291 (2021).

30. H. Tsai, W. Nie, J.-C. Blancon, C. C. Stoumpos, R. Asadpour, B. Harutyunyan, A. J. Neukirch, R. Verduzco, J. J. Crochet, S. Tretiak, L. Pedesseau, J. Even, M. A. Alam, G. Gupta, J. Lou, P. M. Ajayan, M. J. Bedzyk, M. G. Kanatzidis, A. D. Mohite, High-efficiency two-dimensional Ruddlesden–Popper perovskite solar cells. *Nature* **536**, 312–316 (2016).

31. C. Katan, N. Mercier, J. Even, Quantum and dielectric confinement effects in lower-dimensional hybrid perovskite semiconductors. *Chem. Rev.* **119**, 3140–3192 (2019).

32. D. B. Straus, C. R. Kagan, Electrons, excitons, and phonons in two-dimensional hybrid perovskites: Connecting structural, optical, and electronic properties. *J. Phys. Chem. Lett.* **9**, 1434–1447 (2018).

33. J.-C. Blancon, J. Even, C. C. Stoumpos, M. G. Kanatzidis, A. D. Mohite, Semiconductor physics of organic–inorganic 2D halide perovskites. *Nat. Nanotechnol.* **15**, 969–985 (2020).

34. P. Guo, C. C. Stoumpos, L. Mao, S. Sadasivam, J. B. Ketterson, P. Darancet, M. G. Kanatzidis, R. D. Schaller, Cross-plane coherent acoustic phonons in two-dimensional organic-inorganic hybrid perovskites. *Nat. Commun.* **9**, 2019 (2018).

35. A. Giri, A. Z. Chen, A. Mattoni, K. Aryana, D. Zhang, X. Hu, S.-H. Lee, J. J. Choi, P. E. Hopkins, Ultralow thermal conductivity of two-dimensional metal halide perovskites. *Nano Lett.* **20**, 3331–3337 (2020).

36. C. Li, H. Ma, T. Li, J. Dai, M. A. J. Rasel, A. Mattoni, A. Alatas, M. G. Thomas, Z. W. Rouse, A. Shragai, S. P. Baker, B. J. Ramshaw, J. P. Feser, D. B. Mitzi, Z. Tian, Remarkably weak anisotropy in thermal conductivity of two-dimensional hybrid perovskite butylammonium lead iodide crystals. *Nano Lett.* **21**, 3708–3714 (2021).

37. Y. Liu, Y. Zhang, Z. Yang, H. Ye, J. Feng, Z. Xu, X. Zhang, R. Munir, J. Liu, P. Zuo, Q. Li, M. Hu, L. Meng, K. Wang, D.-M. Smilgies, G. Zhao, H. Xu, Z. Yang, A. Amassian, J. Li, K. Zhao, S. Liu, Multi-inch single-crystalline perovskite membrane for high-detectivity flexible photosensors. *Nat. Commun.* **9**, 5302 (2018).

38. S. Li, X. Li, C. A. Kocoj, X. Ji, S. Yuan, E. C. Macropulos, C. C. Stoumpos, F. Xia, L. Mao, M. G. Kanatzidis, P. Guo, Ultrafast excitonic response in two-dimensional hybrid perovskites driven by intense midinfrared pulses. *Phys. Rev. Lett.* **129**, 177401 (2022).

39. Y. Li, A. Chernikov, X. Zhang, A. Rigosi, H. M. Hill, A. M. van der Zande, D. A. Chenet, E.-M. Shih, J. Hone, T. F. Heinz, Measurement of the optical dielectric function of monolayer transition-metal dichalcogenides: MoS_2, $MoSe_2$, WS_2, and WSe_2. *Phys. Rev. B* **90**, 205422 (2014).

40. G. Scuri, Y. Zhou, A. A. High, D. S. Wild, C. Shu, K. De Greve, L. A. Jauregui, T. Taniguchi, K. Watanabe, P. Kim, M. D. Lukin, H. Park, Large excitonic reflectivity of monolayer $MoSe_2$ encapsulated in hexagonal boron nitride. *Phys. Rev. Lett.* **120**, 037402 (2018).

41. P. Guo, W. Huang, C. C. Stoumpos, L. Mao, J. Gong, L. Zeng, B. T. Diroll, Y. Xia, X. Ma, D. J. Gosztola, T. Xu, J. B. Ketterson, M. J. Bedzyk, A. Facchetti, T. J. Marks, M. G. Kanatzidis, R. D. Schaller, Hyperbolic dispersion arising from anisotropic excitons in two-dimensional perovskites. *Phys. Rev. Lett.* **121**, 127401 (2018).

42. O. Yaffe, A. Chernikov, Z. M. Norman, Y. Zhong, A. Velauthapillai, A. van der Zande, J. S. Owen, T. F. Heinz, Excitons in ultrathin organic-inorganic perovskite crystals. *Phys. Rev. B* **92**, 045414 (2015).

43. B. Li, J. Xu, C. A. Kocoj, S. Li, Y. Li, D. Chen, S. Zhang, L. Dou, P. Guo, Dual-hyperspectral optical pump–probe microscopy with single-nanosecond time resolution. *J. Am. Chem. Soc.* **146**, 2187–2195 (2024).

44. S. Li, Z. Dai, L. Li, N. P. Padture, P. Guo, Time-resolved vibrational-pump visible-probe spectroscopy for thermal conductivity measurement of metal-halide perovskites. *Rev. Sci. Instrum.* **93**, 053003 (2022).

45. P. Jiang, X. Qian, R. Yang, Tutorial: Time-domain thermoreflectance (TDTR) for thermal property characterization of bulk and thin film materials. *J. Appl. Phys.* **124**, 161103 (2018).

46. H. Kizuka, T. Yagi, J. Jia, Y. Yamashita, S. Nakamura, N. Taketoshi, Y. Shigesato, Temperature dependence of thermal conductivity of VO_2 thin films across metal–insulator transition. *Jpn. J. Appl. Phys.* **54**, 053201 (2015).

47. D.-W. Oh, C. Ko, S. Ramanathan, D. G. Cahill, Thermal conductivity and dynamic heat capacity across the metal-insulator transition in thin film VO_2. *Appl. Phys. Lett.* **96**, 151906 (2010).

48. A. Boltasseva, H. A. Atwater, Low-loss plasmonic metamaterials. *Science* **331**, 290–291 (2011).

49. P. Guo, R. D. Schaller, J. B. Ketterson, R. P. H. Chang, Ultrafast switching of tunable infrared plasmons in indium tin oxide nanorod arrays with large absolute amplitude. *Nat. Photonics* **10**, 267–273 (2016).

50. A. Agrawal, S. H. Cho, O. Zandi, S. Ghosh, R. W. Johns, D. J. Milliron, Localized surface plasmon resonance in semiconductor nanocrystals. *Chem. Rev.* **118**, 3121–3207 (2018).

51. T. R. Gordon, T. Paik, D. R. Klein, G. V. Naik, H. Caglayan, A. Boltasseva, C. B. Murray, Shape-dependent plasmonic response and directed self-assembly in a new semiconductor building block, indium-doped cadmium oxide (ICO). *Nano Lett.* **13**, 2857–2863 (2013).

52. W.-L. Ong, S. M. Rupich, D. V. Talapin, A. J. H. McGaughey, J. A. Malen, Surface chemistry mediates thermal transport in three-dimensional nanocrystal arrays. *Nat. Mater.* **12**, 410–415 (2013).

53. M. A. Kats, R. Blanchard, P. Genevet, F. Capasso, Nanometre optical coatings based on strong interference effects in highly absorbing media. *Nat. Mater.* **12**, 20–24 (2013).

54. F. Liu, S. Ward, L. Gedvilas, B. Keyes, B. To, Q. Wang, E. Sanchez, S. Wang, Amorphous silicon nitride deposited by hot-wire chemical vapor deposition. *J. Appl. Phys.* **96**, 2973–2979 (2004).

55. W. Paritmongkol, T. Sakurada, W. S. Lee, R. Wan, P. Müller, W. A. Tisdale, Size and quality enhancement of 2D semiconducting metal–organic chalcogenolates by amine addition. *J. Am. Chem. Soc.* **143**, 20256–20263 (2021).

56. W. Li, Z. Wang, F. Deschler, S. Gao, R. H. Friend, A. K. Cheetham, Chemically diverse and multifunctional hybrid organic–inorganic perovskites. *Nat. Rev. Mater.* **2**, 16099 (2017).

57. M. Liu, H. Y. Hwang, H. Tao, A. C. Strikwerda, K. Fan, G. R. Keiser, A. J. Sternbach, K. G. West, S. Kittiwatanakul, J. Lu, S. A. Wolf, F. G. Omenetto, X. Zhang, K. A. Nelson, R. D. Averitt, Terahertz-field-induced insulator-to-metal transition in vanadium dioxide metamaterial. *Nature* **487**, 345–348 (2012).

58. J. D. Caldwell, I. Vurgaftman, J. G. Tischler, O. J. Glembocki, J. C. Owrutsky, T. L. Reinecke, Atomic-scale photonic hybrids for mid-infrared and terahertz nanophotonics. *Nat. Nanotechnol.* **11**, 9–15 (2016).

59. S. Dai, Q. Ma, M. K. Liu, T. Andersen, Z. Fei, M. D. Goldflam, M. Wagner, K. Watanabe, T. Taniguchi, M. Thiemens, F. Keilmann, G. C. A. M. Janssen, S. E. Zhu, P. Jarillo-Herrero, M.

M. Fogler, D. N. Basov, Graphene on hexagonal boron nitride as a tunable hyperbolic metamaterial. *Nat. Nanotechnol.* **10**, 682–686 (2015).

60. C. Wang, S. Huang, Q. Xing, Y. Xie, C. Song, F. Wang, H. Yan, Van der Waals thin films of WTe_2 for natural hyperbolic plasmonic surfaces. *Nat. Commun.* **11**, 1158 (2020).

61. X. Ye, J. Fei, B. T. Diroll, T. Paik, C. B. Murray, Expanding the spectral tunability of plasmonic resonances in doped metal-oxide nanocrystals through cooperative cation–anion codoping. *J. Am. Chem. Soc.* **136**, 11680–11686 (2014).

62. B. T. Diroll, T. R. Gordon, E. A. Gaulding, D. R. Klein, T. Paik, H. J. Yun, E. D. Goodwin, D. Damodhar, C. R. Kagan, C. B. Murray, Synthesis of n-type plasmonic oxide nanocrystals and the optical and electrical characterization of their transparent conducting films. *Chem. Mater.* **26**, 4579–4588 (2014).

63. R. W. Millar, The heat capacity at low temperatures of zinc oxide and of cadmium oxide. *J. Am. Chem. Soc.* **50**, 2653–2656 (1928).

64. L. Lindsay, D. S. Parker, Calculated transport properties of CdO: Thermal conductivity and thermoelectric power factor. *Phys. Rev. B* **92**, 144301 (2015).

65. F. O. Cedeño, M. M. Prieto, J. Xiberta, Measurements and estimate of heat capacity for some pure fatty acids and their binary and ternary mixtures. *J. Chem. Eng. Data* **45**, 64–69 (2000).

66. S. Li, Z. Dai, C. A. Kocoj, E. I. Altman, N. P. Padture, P. Guo, Photothermally induced, reversible phase transition in methylammonium lead triiodide. *Matter* **6**, 460–474 (2023).

67. D. K. Efetov, R.-J. Shiue, Y. Gao, B. Skinner, E. D. Walsh, H. Choi, J. Zheng, C. Tan, G. Grosso, C. Peng, J. Hone, K. C. Fong, D. Englund, Fast thermal relaxation in cavity-coupled graphene bolometers with a Johnson noise read-out. *Nat. Nanotechnol.* **13**, 797–801 (2018).

68. J. Shi, D. Yoo, F. Vidal-Codina, C.-W. Baik, K.-S. Cho, N.-C. Nguyen, H. Utzat, J. Han, A. M. Lindenberg, V. Bulović, M. G. Bawendi, J. Peraire, S.-H. Oh, K. A. Nelson, A room-temperature polarization-sensitive CMOS terahertz camera based on quantum-dot-enhanced terahertz-to-visible photon upconversion. *Nat. Nanotechnol.* **17**, 1288–1293 (2022).

69. C. Chen, C. Li, S. Min, Q. Guo, Z. Xia, D. Liu, Z. Ma, F. Xia, Ultrafast silicon nanomembrane microbolometer for long-wavelength infrared light detection. *Nano Lett.* **21**, 8385–8392 (2021).
